# Long noncoding RNA SGO1-AS1 inactivates TGFβ signaling by facilitating TGFB1/2 mRNA decay and inhibits gastric carcinoma metastasis

**DOI:** 10.1186/s13046-021-02140-0

**Published:** 2021-10-28

**Authors:** Donglan Huang, Ke Zhang, Wenying Zheng, Ruixin Zhang, Jiale Chen, Nan Du, Yuanyuan Xia, Yan Long, Yixue Gu, Jianhua Xu, Min Deng

**Affiliations:** 1 Affiliated Cancer Hospital & Institute of Guangzhou Medical University, No.78 Hengzhigang Road, Guangzhou, 510095 Guangdong China; 2 Department of Clinical Laboratory, Sun Yat-Sen University Cancer Center, State Key Laboratory of Oncology in South China, Guangzhou, China; 3 Department of Oncology, The Fifth Affiliated Hospital, Guangzhou Medical University, Guangzhou, China; 4 Department of Laboratory, Guangzhou Women and Children’s Medical Centre, Guangzhou Medical University, Guangzhou, China; 5 Laboratory of Oncology Science and Molecular Biology, ShunDe Hospital of Guangzhou University of Chinese Medicine, No.12 Jinsha Avenue, Shunde District, Foshan, 528333 Guangdong China

**Keywords:** Gastric carcinoma, Metastasis, lncRNA, SGO1-AS1, TGFβ, ZEB1

## Abstract

**Background:**

Although thousands of long noncoding RNAs (lncRNAs) have been annotated, only a few lncRNAs have been characterized functionally. In this study, we aimed to identify novel lncRNAs involved in the progression of gastric carcinoma (GC) and explore their regulatory mechanisms and clinical significance in GC.

**Methods:**

A lncRNA expression microarray was used to identify differential lncRNA expression profiles between paired GCs and adjacent normal mucosal tissues. Using the above method, the lncRNA SGO1-AS1 was selected for further study. Quantitative reverse transcription polymerase chain reaction (qRT-PCR) and in situ hybridization (ISH) were performed to detect SGO1-AS1 expression in GC tissues. Gain-of-function and loss-of-function analyses were performed to investigate the functions of SGO1-AS1 and its upstream and downstream regulatory mechanisms in vitro and in vivo.

**Results:**

SGO1-AS1 was downregulated in gastric carcinoma tissues compared to adjacent normal tissues, and its downregulation was positively correlated with advanced clinical stage, metastasis status and poor patient prognosis. The functional experiments revealed that SGO1-AS1 inhibited GC cell invasion and metastasis in vitro and in vivo. Mechanistically, SGO1-AS1 facilitated TGFB1/2 mRNA decay by competitively binding the PTBP1 protein, resulting in reduced TGFβ production and, thus, preventing the epithelial-to-mesenchymal transition (EMT) and metastasis. In addition, in turn, TGFβ inhibited SGO1-AS1 transcription by inducing ZEB1. Thus, SGO1-AS1 and TGFβ form a double-negative feedback loop via ZEB1 to regulate the EMT and metastasis.

**Conclusions:**

SGO1-AS1 functions as an endogenous inhibitor of the TGFβ pathway and suppresses gastric carcinoma metastasis, indicating a novel potential target for GC treatment.

**Supplementary Information:**

The online version contains supplementary material available at 10.1186/s13046-021-02140-0.

## Background

Gastric carcinoma is one of the most common malignancies worldwide and the fourth leading cause of cancer death [1]. Approximately 40% of patients with gastric carcinoma present with metastases, and only approximately 5% of these patients exhibit 5-year survival [2]. The prognosis of GC patients with metastatic disease remains poor due to the lack of effective therapies. New therapeutic options will become available only if we improve our understanding of the mechanisms underlying metastatic spread.

LncRNAs are transcripts longer than 200 nucleotides without protein-coding potential [3]. Tens of thousands of lncRNAs are expressed in human cells, but the function of most lncRNAs remains unknown [4]. An increasing number of studies have demonstrated the importance of lncRNAs for regulating a wide range of processes, including development, differentiation, cell proliferation, cell death and cancer development [5, 6]. Recently, several GC-implicated lncRNAs have been identified, and their functions and mechanisms have been clarified [7–11]. For instance, the lncRNA GClnc1 promotes gastric carcinogenesis and may act as a scaffold for WDR5 and KAT2A complexes to specify the histone modification pattern [9]. The lncRNA GMAN enhances the translation of ephrin A1 mRNA by competitively binding GMAN-AS and, thus, promotes GC invasion and metastasis [10]. However, the well-characterized lncRNAs involved in GC are merely the tip of the iceberg, and an even larger number remain unknown.

Here, we demonstrate that the lncRNA SGO1-AS1 (also known as SGOL1-AS1), which is downregulated in gastric carcinoma and associated with tumor progression and patient prognosis, prevents gastric carcinoma EMT, invasion and metastasis in vitro and in vivo. Mechanistically, SGO1-AS1 reduces the stability of TGFB1/2 mRNA by competitively binding the PTBP1 protein, resulting in reduced TGFβ production. In turn, TGFβ inhibits SGO1-AS1 transcription by inducing ZEB1. Thus, in this study, we identified a novel metastasis-suppressive lncRNA, i.e., SGO1-AS1, with crucial biological, mechanistic and clinical impacts on GC that mediates a double-negative feedback loop with TGFβ via ZEB1.

## Methods

### Clinical specimens

Five pairs of snap-frozen GC tissues and matched adjacent normal mucosa tissues were obtained for the lncRNA microarray analysis. Furthermore, the following two cohorts of frozen samples were collected for the qRT-PCR assay: a small GC cohort (Cohort 1) containing 18 pairs of GC tissues and corresponding adjacent normal mucosa tissues to confirm 13 lncRNAs with more than a 4-fold difference in the microarray analysis and a large GC cohort (Cohort 2) including 92 pairs of GC tissues and matched adjacent normal samples to detect the expression levels of SGO1-AS1, TGFB1/2 and ZEB1. Additionally, GC tissue microarrays containing 95 GC tissues and 80 adjacent tissues (Cohort 3) were included in this study for the ISH analysis. All tissues were collected immediately after surgery from the Affiliated Cancer Hospital of Guangzhou Medical University (Guangzhou, Guangdong, China). All procedures carried out in this research involving human participants were performed in accordance with the ethical standards of the Institutional Review Board of the Affiliated Cancer Hospital of Guangzhou Medical University. The clinical and histopathological characteristics of the patients are described in Additional file 1: Table S1–2.

### Microarray analysis

The total RNA was extracted from 5 paired GC tissues and corresponding adjacent normal mucosa tissues using TRIzol reagent (Invitrogen, Carlsbad, CA, USA) according to the manufacturer’s instructions. The total RNA was amplified and reverse-transcribed into fluorescent cDNA. Then, the labeled cDNA was hybridized onto the LncRNA+mRNA Human Gene Expression Microarray V4.0 (Agilent, Palo Alto, CA), and after washing, the arrays were scanned with an Agilent Scanner G2565CA (Agilent). Agilent Feature Extraction software (version 10.7.3.1) was used to analyze the acquired array images and the Agilent qRT-PCR results. The data are available via Gene Expression Omnibus (GEO) under accession number GSE157289.

### qRT-PCR

The total RNA was isolated from patient tissues and cultured cells using TRIzol reagent (Invitrogen), and cDNA was synthesized using a PrimeScript RT Reagent Kit (Takara, Otsu, Japan). Subsequently, quantitative polymerase chain reaction (qPCR) analyses were performed using a SYBR Premix Ex Taq Kit (Applied Biosystems, Foster City, CA, USA). β-actin was used as the endogenous control to normalize gene expression. The mRNA expression of SGO1-AS1, TGFB1, TGFB2 and PTBP1 in the human tissues is presented as -∆Ct, and the gene expression in cells with different treatments is presented as 2^-∆∆Ct^. The ∆Ct was calculated by subtracting the Ct of β-actin from the Ct of the gene of interest. The ∆∆Ct was calculated by subtracting the ∆Ct of the control sample from the ∆Ct of the treatment sample. The primer sequences for each gene are provided in Additional file 1: Table S3.

### In situ hybridization (ISH)

The ISH analysis was performed using a kit from Boster (Wuhan, Hubei, China). Tissue microarray slides were deparaffinized, digested with proteinase K, hybridized with DIG-labeled probes for SGO1-AS1 and U6 (positive control) at 52 °C overnight and subsequently visualized with an anti-DIG-POD antibody and DAB complex. The SGO1-AS1 probe was 5′-CCGCCTCCCAGCCAACCAATGGAGGAGCGAGGCG-3′. The results were evaluated by two individuals in a blinded fashion, and the SGO-AS1 expression levels were quantified according to its positive percentage and staining intensity.

### Rapid amplification of cDNA ends (RACE) analysis

We used 5′-RACE and 3′-RACE analyses to determine the transcriptional initiation and termination sites of SGO1-AS1 using a SMARTer™ RACE cDNA Amplification Kit (Clontech, Palo Alto, CA, USA) following the manufacturer’s instructions. Nested PCR products were cloned into the pMD20-T vector and then sequenced. The sequences of the SGO1-AS1-specific primers used in the nested PCR of the RACE assay are shown in Additional file 1: Table S4.

### Subcellular fractionation

Nuclear and cytoplasmic separation was performed using a PARIS Kit (Life Technologies, USA) according to the manufacturer’s instructions, and then, a qRT-PCR analysis was conducted.

### Cell culture

The GC cell lines SGC7901, BGC823, AGS, MGC803, MKN45 and MKN28 were obtained from the Chinese Academy of Medical Science (Beijing, China), and the gastric epithelial cell line GES-1 was obtained from the Beijing Institute for Cancer Research (Beijing, China). The GC cell line NCI-N87 and the HEK293T cell line were obtained from the American Type Culture Collection (Manassas VA, USA). The cell lines involved in our experiments were reauthenticated by a short tandem repeat analysis every 6 months after resuscitation in our laboratory. These cells were cultured in Dulbecco’s modified Eagle’s medium (DMEM, Gibco, USA) supplemented with 10% fetal bovine serum (Gibco) at 37 °C in 5% CO_2_.

### RNAi, plasmid construction and cell transfection

The recombinant lentiviral vectors used for SGO1-AS1 overexpression or knockdown were purchased from RiboBio (Guangzhou, Guangdong, China), and the PTBP1 short hairpin RNA (shRNA) lentiviral vectors were obtained from GeneChem (Shanghai, China). The target sequences for SGO1-AS1 and PTBP1 were as follows: shAS1#1, 5′-GCTATCTTCCTCCTCCTCACA-3′; shAS1#2, 5′-CTACCGCCGCCACATTCGAAA-3′; shAS1#3, 5′-GCCTCCCTCTTGTGAGAAGAA-3′; shAS1#4, 5′-AGCTTGCAACGCGGAAGCAGC-3′; and shPTBP1, 5′-GCGGCCAGCC CATCTACATC-3′. To establish the cell lines that stably overexpress or deplete SGO1-AS1, SGC-7901 cells were infected with recombinant SGO1-AS1 lentiviruses, while MKN28 cells were infected with SGO1-AS1 shRNA lentiviruses. Then, the infected cells were selected with 1 mg/L puromycin (InvivoGen, San Diego, CA, USA) for 2 weeks to obtain cells with stable overexpression or knockdown of SGO1-AS1. siRNAs targeting ZEB1 or AGO2 were designed and synthesized by Sangon Biotech (Shanghai, China), and their sequences are as follows: siZEB1#1 sense, GGCAAGUGUUGGAGAAUAAUC, antisense, UUAUUCUCCAACACUUGCCUU; siZEB1#2 sense, GGACAGCACAGUAAAUCUACA, antisense, UAGAUUUACUGUGCUGUCCUG; siAGO2 sense, GGUUGAUACUUAAGCUCUAUU, antisense, UAGAGCUUAAGUAUCAACCUG. To construct the reporter vectors for SGO1-AS1 promoter activity, the wild-type SGO1-AS1 promoter sequence (1 kb sequence upstream of the transcription start site) and its ZEB1-binding site mutated sequences were chemosynthesized by Huada (Shenzhen, Guangdong, China) and inserted into the vector pGL3 basic (Promega) upstream of the firefly luciferase gene.

### PTBP1 knockout by CRISPR/Cas9

A small guide RNA (sgRNA) targeting the genome sequence of PTBP1 was cloned into LentiCRISPRv2 (Addgene), and lentivirus particles were generated by cotransfecting the recombinant vector and packaging plasmids into HEK293T packaging cells. MKN28 cells were infected with lentiviruses, and single cells were isolated 48 h after infection by FACS (BD FACS Aria III) into 96-well plates. Independent clones were allowed to grow for 3 weeks. The PTBP1 knockout cells were identified by Western blotting and targeted Sanger sequencing. The sgRNA targeting PTBP1 was 5′-CAGAGCAGACCCGCGGGGGA-3′.

### Western blotting analysis

The Western blotting analysis was performed using standard procedures. The following primary antibodies were used in the experiments: anti-PTBP1 antibody (Cell Signaling Technology, Beverly, MA, USA), anti-PTBP2 antibody (Abcam, Cambridge, UK), anti-PTBP3 antibody (Sigma-Aldrich, St. Louis, MO, USA), anti-HNRNPK antibody (Abcam), anti-HNRNPM antibody (Sigma-Aldrich), anti-FUBP3 antibody (Abcam), anti-CPSF2 antibody (Abcam), anti-G3BP2 antibody (Atlas Antibodies), anti-TGFβ1 antibody (Proteintech Group), anti-TGFβ2 antibody (Abcam), anti-p-SMAD2 antibody (Cell Signaling Technology), anti-SMAD2 antibody (Cell Signaling Technology), anti-p-SMAD3 antibody (Cell Signaling Technology), anti-SMAD3 antibody (Cell Signaling Technology), anti-SMAD5 antibody (Abcam), anti-ID2 antibody (Abcam), anti-ZEB1 antibody (Abcam), anti-SNAI antibody (Abcam), anti-E-cadherin antibody (Proteintech Group), anti-Vimentin antibody (Cell Signaling Technology), anti-N-cadherin antibody (Cell Signaling Technology) and anti-GAPDH antibody (Sigma-Aldrich). The blots were incubated with a goat anti-rabbit or anti-mouse secondary antibody (Sigma-Aldrich) and visualized with a commercial ECL kit (Pierce, Rockford, IL).

### RNA pull-down assay

The RNA pull-down assays were carried out as previously described. Briefly, the SGO1-AS1 sequences were cloned into the pMD20-T vector with the T7 promoter and transcribed in vitro with biotin RNA labeling mix and T7 RNA polymerase (Invitrogen) according to the manufacturer’s instructions. The RNA pulldown assay was performed using a Pierce Magnetic RNA-Protein Pull-Down Kit (Millipore, Bedford, MA, USA) according to the manufacturer’s instructions. Finally, the retrieved proteins were measured using sodium dodecyl sulfate-polyacrylamide gel electrophoresis (SDS PAGE) gels for mass spectrometry or a Western blot analysis.

### RNA immunoprecipitation (RIP) assay

The RIP assays were performed using a Magna RIP RNA-Binding Protein Immunoprecipitation Kit (Millipore, Bedford, MA, USA) according to the manufacturer’s instructions. Briefly, 100 μL of cell extract were incubated with magnetic bead-antibody complex. Antibodies were used for RIP, and IgG served as a negative control. The precipitated RNAs were isolated using TRIzol (Invitrogen) for the RNA sequencing (RNA-seq) and qRT-PCR analyses.

### Chromatin immunoprecipitation (ChIP) assay

The ChIP assays were performed using a Chromatin Immunoprecipitation Assay Kit (Millipore, Bedford, MA, USA). MKN28 cells were exposed to TGFβ1 or vehicle for 24 h and then crosslinked, lysed and sonicated. Immunoprecipitation was performed using an anti-ZEB1 antibody (Abcam, Cambridge, UK) and IgG. The precipitated DNA was quantified using qPCR and normalized to the respective 2% input.

### RNA-seq analysis

To identify the differentially expressed genes upon PTBP1 knockout, the total RNA was isolated from the PTBP1 knockout or control MKN28 cells using TRIzol reagent, and PolyA RNA was subsequently purified from the total RNA using the NEBNext Poly(A) mRNA Magnetic Isolation Module. RNA-seq was performed to detect the mRNA expression profiles at GENTED (Shanghai, China) using HiSeq3000 (Illumina, USA). The differentially expressed genes with a fold change > 2 and a *P*-value < 0.05 were selected. To reveal the PTBP1-bound mRNAs, RIP experiments were conducted using a PTBP1 antibody (Cell Signaling Technology) or IgG. The total RNA was isolated with TRIzol (Invitrogen), and ribosomal RNA was removed from the total RNA. RNA-seq was performed at CLOUDSEQ (Shanghai, China) using HiSeq3000 (Illumina, USA). The data are available via GEO under accession numbers GSE157582 and GSE157941.

### Luciferase reporter assay

HEK293T cells were seeded in 24-well plates and transfected with SGO1-AS1 promoter reporter constructs with wild-type or mutated ZEB1 binding sites. The pTK-Cluc vector was used as an internal transfection control. The transfected cells were treated with TGFβ1 (5 ng/mL) or vehicle control for 48 h, and firefly and Renilla luciferase activities were measured using a Dual-Luciferase Reporter Assay System (Promega) following the manufacturer’s instructions. The SBE4 promoter luciferase reporter vector (Addgene) was transfected into the PTBP1 knockout or control MKN28 cells. In addition, HEK293T cells were transfected with SBE4 promoter reporter vectors and then treated with conditioned medium from cells with SGO1-AS1 knockdown or overexpression. The firefly and Renilla luciferase activities were measured 48 h after the transfection using a dual luciferase system.

### Cell invasion, migration and proliferation assays

For the cell invasion assay, starved cells suspended in serum-free DMEM were seeded into the upper chamber with Matrigel in the insert of a 24-well culture plate (Corning Costar). Medium containing 15% fetal bovine serum was added to the lower compartment as a chemoattractant. After incubation for 48 h, the invasive cells adhering to the lower membrane of the inserts were fixed, stained, counted and imaged. The cell migration ability was measured using a wound-healing assay. The cells were placed in 6-well plates and cultured until reaching 90% confluence. An artificial scratch was created using a 10 μL pipette tip, and the cells were cultured in serum-free medium for 36 h or 48 h. Wound closure images were captured in the same field under magnification. Cell proliferation was examined using cell counting. The cells were seeded into 6-well plates, and the cell numbers were counted after 1, 2, 3, 4, 5, 6 and 7 days of culture in DMEM supplemented with 10% fetal bovine serum using a Coulter Counter.

### Sphere culture

Cells were seeded into ultralow attachment 6-well plates (Corning Costar) and cultured in DMEM/F12 medium (Gibco) supplemented with 2% B27 (Life Technologies), 20 ng/ml FGF (R&D Systems, MN, USA), 20 ng/ml EGF (R&D Systems) and 5 μg/ml insulin (R&D Systems). Two weeks later, sphere pictures were obtained, and the sphere formation ratios were calculated.

### Animal experiments

Subsequently, 6- to 8-week-old female BALB/c nude mice were purchased from the Experimental Animal Center of Guangdong (Foshan, Guangdong, China). To investigate the role of SGO1-AS1 in tumor metastasis and growth in vivo, luciferase-labeled SGC7901 cells overexpressing SGO1-AS1 or the control vector (2 × 10^6^ cells per mouse) were injected into the tail vein or stomach of the BALB/c nude mice. The luciferase signal intensity was monitored in vivo using an In Vivo Imaging System (FX PRO, Bruker, Billerica, MA, USA). Then, the mice were sacrificed, and the metastatic foci in the abdominal cavity and lung were evaluated. In addition, SGC7901 cells with SGO1-AS1 overexpression or control cells were subcutaneously injected into nude mice The mice were sacrificed 28 days after implantation, and the tumors were excised and weighed.

To confirm the inhibitory effects of SGO1-AS1 on metastasis activity via TGFβ signaling in vivo, we orthotopically implanted luciferase-labeled MKN28 cells stably expressing shSGO1-AS1 or control shRNA into the stomach of nude mice and treated the mice with saline or SB431542 (20 mg/kg body weight, i.p.) three times per week for 3 weeks. The luciferase signal intensity was monitored in vivo by bioluminescence imaging. All animal studies were approved by the Institutional Animal Care and Use Committee of Guangzhou Medical University, and the animals were treated ethically and humanely.

### Statistical analysis

A Student’s t-test or chi-square test was used for the two-sample comparisons. The differences among three or more groups were analyzed with a two-way analysis of variance. The overall survival curves were plotted using the Kaplan-Meier method, and the survival differences were evaluated with a log-rank test. A Cox regression was utilized to estimate the hazard ratio and 95% confidence intervals of survival. The pairwise expression correlations were analyzed using Pearson correlation tests. *P-values* < 0.05 were considered statistically significant.

## Results

### SGO1-AS1 is downregulated in gastric carcinoma tissues and inversely associated with tumor progression

**Fig. 1:**
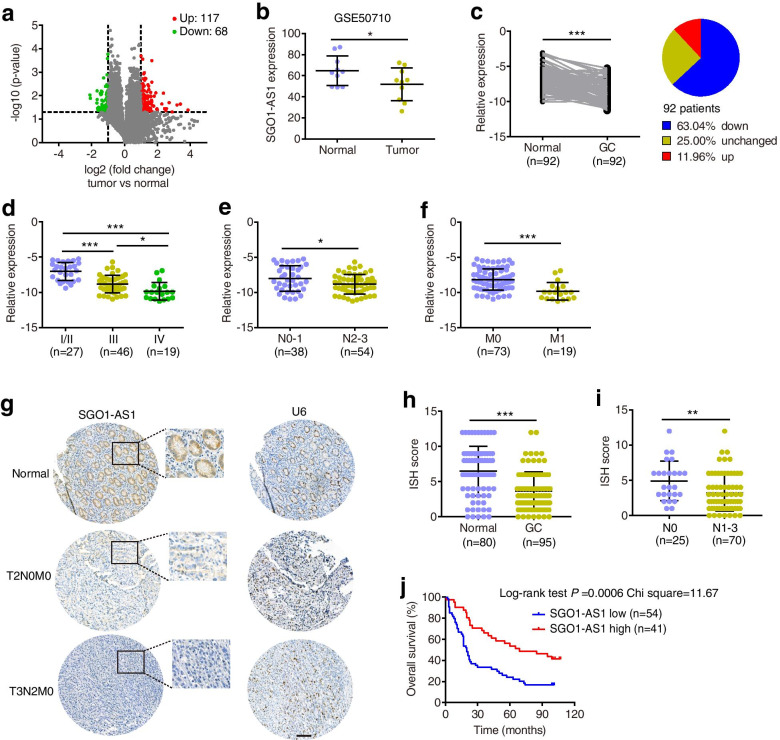
SGO1-AS1 is downregulated in GC tissues and associated with GC progression. **a.** Volcano plots of differentially expressed lncRNAs in GCs vs. matched normal tissues shown in green and red, respectively. **b.** SGO1-AS1 expression levels in 10 pairs of GCs and adjacent normal tissues from the GEO dataset (GSE50710). **c.** Relative expression levels of SGO1-AS1 in 92 paired GC and normal tissues from Cohort 2 patients were quantified by qRT-PCR. SGO1-AS1 was downregulated (> 2-fold) in 63% (58 of 92) of the GC tissues (tumor) relative to that in the adjacent noncancerous tissues (normal). **d-f.** Assessment of the SGO1-AS1 expression levels in GCs according to their clinical stage **(d)** and status of lymph node **(e)** or distant metastasis **(f)** based on a qPCR analysis of GC. **g-i**. RNA ISH analyses of SGO1-AS1 expression in 95 GC specimens and 80 normal tissues on tissue microarrays. Scale bar: 50 μm. **j.** Kaplan-Meier analyses of the overall survival of patients with GCs (*n* = 95) based on the SGO1-AS1 expression levels. The defined high and low expression levels of SGO1-AS1 were stratified according to the median expression level. Error bars indicate the standard deviation (SD). *P < 0.05, **P < 0.01, ***P < 0.001

To identify GC-relevant lncRNAs, we examined the lncRNA expression profiles in five paired GC and adjacent normal mucosa tissues using a microarray. We found that 185 lncRNAs were differentially expressed in GCs compared to those in the adjacent tissues (fold change > 2 and *P* < 0.05, Fig. 1a); 13 of these lncRNAs were upregulated or downregulated by more than fourfold. We selected these lncRNAs with more than fourfold differences for qPCR expression validation in a small GC cohort (18 pairs of GC and adjacent normal tissues, Cohort 1). Among these lncRNAs, SGO1-AS1 was the most differentially expressed in GC relative to the normal samples (Additional file 1: Fig. S1). Furthermore, SGO1-AS1 was also downregulated in GC tissues in one publicly published dataset (GSE50710) [12] in the GEO database (Fig. 1b).

The SGO1-AS1 gene has two annotated transcripts in the Genecode database (GENCODE V23, Additional file 1: Fig. S2a). Nevertheless, the expression of the short isoform was not detected in any GC cell lines, GC tissues, or normal samples in this study (data not shown), but the long isoform was expressed to varying degrees in the GC tissues, normal samples and GC cell lines (Fig. 1c, and Additional file 1: Fig. S3a). Therefore, we focused on the long isoform of SGO1-AS1 in our further analyses. For convenience, we refer to this isoform as SGO1-AS1. In 5′ and 3′ RACE, SGO1-AS1 was revealed to be a 1392-nucleotide antisense transcript (Additional file 1: Fig. S2b) with a sequence that is partially complementary to SGO1 mRNAs. We examined the coding capability of SGO1-AS1 using the Coding-Potential Assessment Tool (CPAT) [13] and the Coding Potential Calculator (CPC) [14]. The results showed that SGO1-AS1 has no protein-coding potential (Additional file 1: Fig. S2c). Furthermore, SGO1-AS1 was mainly located in the cytoplasm of the normal and GC cells as shown by the qRT-PCR analysis with nuclear/cytoplasmic RNA fractionation (Additional file 1: Fig. S2d) and RNA ISH analysis (Fig. 1g).

In another cohort of 92 pairs of GC and adjacent normal tissues (Cohort 2), we further confirmed the downregulation of SGO1-AS1 in GC tissues via a qRT-PCR analysis, and 63% (58/92) of the GC cases showed more than 2-fold downregulation of SGO1-AS1 relative to the corresponding normal tissues (Fig. 1c). Moreover, decreased levels of SGO1-AS1 were correlated with clinical stage, lymph node metastasis and distant metastasis (Fig. 1d-f). This result was further confirmed by ISH analyses of 80 cases of normal gastric mucosa tissues and 95 GC tumor tissues (Cohort 3) in tissue microarrays (Fig. 1g-i, and Additional file 1: Table S5). Moreover, the survival analysis showed that low levels of SGO1-AS1 expression in GC tissues were associated with unfavorable overall survival for GC patients (log rank Chi square = 11.67, *P* = 0.0006, Fig. 1i). Simultaneously, the Cox proportional hazards regression analysis indicated that low SGO1-AS1 expression was an independent predictor of GC prognosis (Additional file 1: Table S6). Taken together, these results demonstrate a reverse correlation between SGO1-AS1 expression and GC progression.

### SGO1-AS1 suppresses gastric carcinoma cell invasion and metastasis

**Fig. 2:**
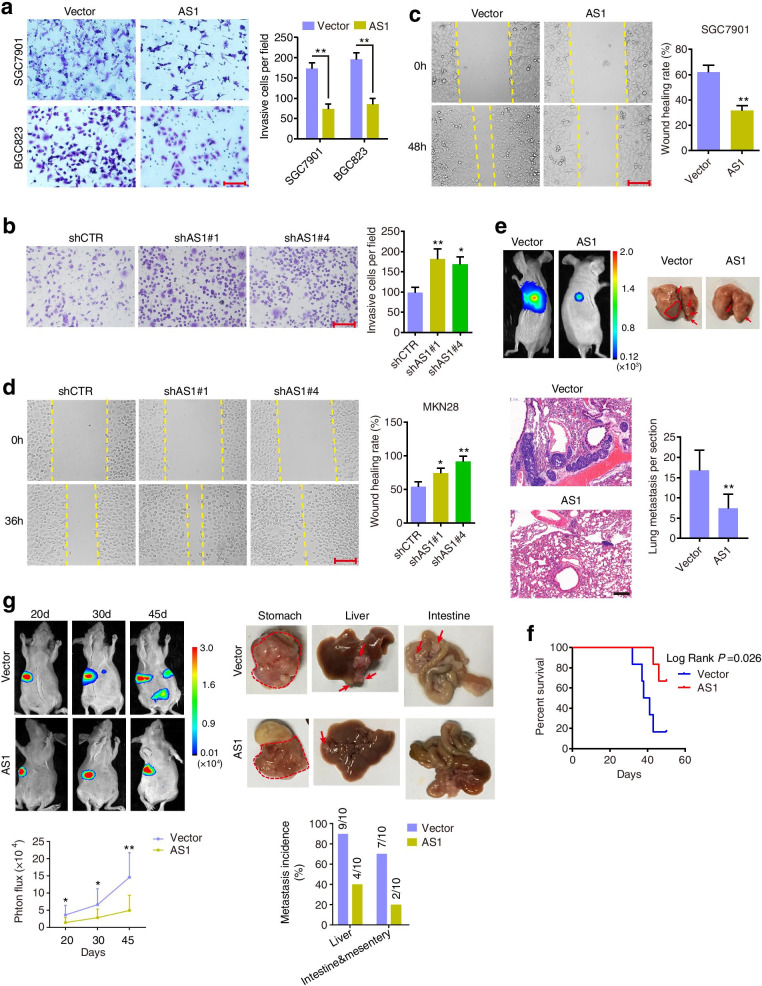
SGO1-AS1 suppresses the invasive and metastatic activity of GC cells in vitro and in vivo. **a-b.** Transwell assay measuring the invasion of SGC7901 and BGC823 cells stably expressing SGO1-AS1 **(a)**, MKN28 cells stably silencing SGO1-AS1 **(b)** and their respective control cells. **c-d.** Wound healing assay measuring the migratory ability of the indicated GC cells. **a-d** Scale bars, 150 μm. Error bars, SD from three independent experiments performed in triplicate. *P < 0.05, **P < 0.01, ***P < 0.001. **e.** SGC7901 cells stably expressing SGO1-AS1 or vector were intravenously injected into the tail vein of nude mice. Representative bioluminescent images and H&E-stained lung sections of the mice are shown. The number of metastatic foci was quantified. Scale bar, 200 μm. Error bars represent SD (*n* = 5 mice/group). Arrows or circles indicate metastatic nodules. **P < 0.01. **f.** Kaplan-Meier survival curve of mice in a parallel experiment. **g.** SGO1-AS1-overexpressing SGC7901 cells and control cells were orthotopically injected into the stomach of nude mice (10 mice per group). The mice were sacrificed 45 days later, and the tumor nodules in the abdominal cavity were examined. Representative IVIS luciferase in vivo images and bright views of livers and intestines isolated from the mice are shown. The bioluminescence signal and number of mice with metastasis were quantified. Arrows or circles indicate metastatic nodules. **P < 0.01

Given the inverse relationship between the SGO1-AS1 expression level and GC progression, we investigated whether SGO1-AS1 could affect GC cell invasion and metastasis. Therefore, we first tested the endogenous expression levels of SGO1-AS1 in gastric cell lines and found it to be expressed at low levels in the SGC7901, BGC823 and MGC803 cells and at relatively high levels in the MKN28 cells (Additional file 1: Fig. S3a). Therefore, SGC7901 and BGC823 cell lines were selected to stably overexpress SGO1-AS1, and MKN28 cell line was chosen to stably deplete SGO1-AS1 with effective shRNAs using a lentiviral system (Additional file 1: Fig. S3b, c). The results from the Transwell and wound-healing assays showed that stable SGO1-AS1 overexpression repressed the migration and invasion of SGC7901 and BGC823 cells (Fig. 2a, c and Additional file 1: Fig. S3d). In contrast, knockdown of SGO1-AS1 by two different shRNAs significantly enhanced the migration and invasion activities of MKN28 cells (Fig. 2b, d). Moreover, SGO1-AS1 inhibited long-term cell growth but had no significant impact on short-term growth (Additional file 1: Fig. S3e). In addition, the soft agar colony formation assays revealed that SGO1-AS1 overexpression markedly reduced the colony number and size, while silencing SGO1-AS1 had the opposite effect (Additional file 1: Fig. S3f, g).

In our subsequent in vivo study, SGC7901 cells stably expressing empty vector or SGO1-AS1 were injected into the tail vein of nude mice, and the formation of pulmonary metastases was measured. Overexpression of SGO1-AS1 reduced the ability of SGC7901 cells to form lung metastases in the mice (Fig. 2e). Moreover, the survival time of the mice injected with SGC7901 cells was prolonged when SGO1-AS1 was overexpressed (Fig. 2f). In addition, SGC7901 cells with stable expression of SGO1-AS1 or empty vector were injected into the corpus of the stomach of nude mice. The metastasis signals observed in the SGO1-AS1-overexpressing group were lower than those observed in the control group by bioluminescence imaging (Fig. 2g). Then, the mice were sacrificed, and the metastatic foci in the abdominal cavity were evaluated. We found that 90% (9/10) of the control mice, but only 40% of the mice in the SGO1-AS1-overexpressing group, had metastatic nodules in the liver (Fig. 2g). In addition, 70% (7/10) of the mice in the control group and only 20% (2/10) of the mice in the SGO1-AS1-overexpressing group had intestinal and mesenteric metastases (Fig. 2g). Subcutaneous xenografts were also established in nude mice using SGC7901 cells with stable expression of empty vector or SGO1-AS1. The overexpression of SGO1-AS1 moderately inhibited tumor growth (Additional file 1: Fig. S3h). Taken together, these findings demonstrate that SGO1-AS1 inhibits GC invasion and metastasis both in vitro and in vivo.

### SGO1-AS1 is associated with PTBP1

**Fig. 3:**
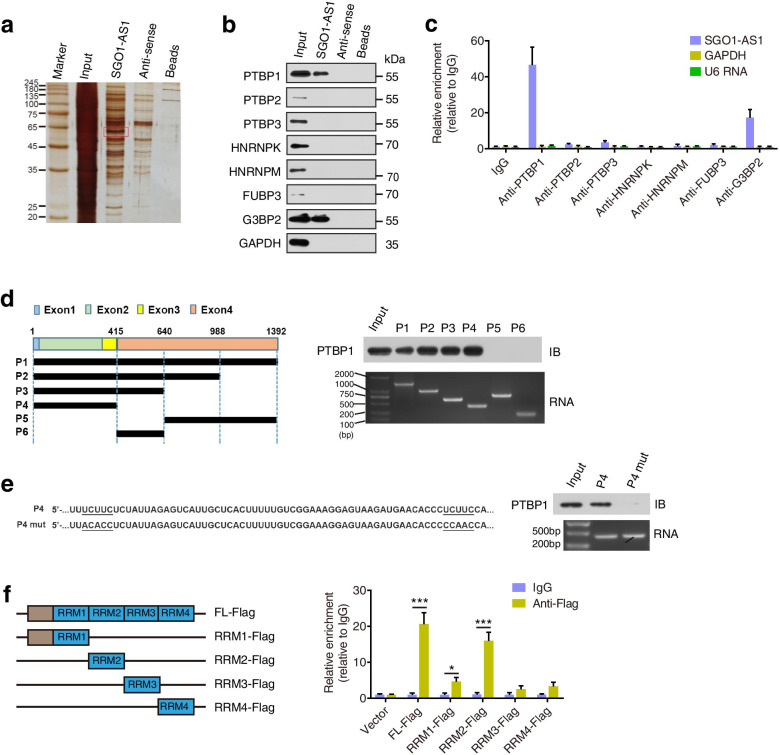
SGO1-AS1 RNA interacts with the PTBP1 protein. **a.** Identification of SGO1-AS1-associated proteins by an RNA pulldown assay. The proteins pulled down by SGO1-AS1 or the antisense RNA of SGO1-AS1 incubated with SGC7901 cell extracts were resolved by SDS-PAGE and subjected to silver staining. A specific band was identified in the SGO1-AS1 group and is marked with a red box. **b.** Western blot analysis validation of the biotin-labeled RNA pulldown assay using sense or antisense probes in SGC7901 cells. GAPDH was used as a negative control. **c.** RIP-qPCR detection of the indicated RNAs retrieved by specific antibodies in MKN28 cells. The fold enrichment of SGO1-AS1 relative to IgG was determined by qRT-PCR. U6 RNA and GAPDH were used as negative controls. **d.** Serial deletions of SGO1-AS1 were used in the RNA pulldown assays to identify the core regions of SGO1-AS1 required for a physical interaction with PTBP1. Left panel: graphic illustration of the SGO1-AS1 probes. **e.** RNA pulldown assay was performed using biotin-labeled 1–415 nt (P4) and PTBP1-binding site mutated RNA (P4 mut) of SGO1-AS1, followed by a Western blot analysis. **f.** RIP assays were performed using an anti-Flag antibody in HEK293T cells transfected with Flag-tagged PTBP1 or its deletion mutants. qRT-PCR was used to measure the enrichment of SGO1-AS1. Error bars represent SD. *P < 0.05, ***P < 0.001

Subsequently, we explored the molecular mechanism underlying the SGO1-AS1-induced inhibition of metastasis. SGO1-AS1 is an antisense transcript that partially overlaps the coding gene SGO1 (or SGOL1). Therefore, we examined whether SGO1-AS1 could affect the expression of the sense gene SGO1 and found that although knockdown of SGO1-AS1 moderately induced SGO1 expression, overexpression of SGO1-AS1 did not affect the expression of SGO1 (Additional file 1: Fig. S4a, b). Then, we identified potential SGO1-AS1-interacting proteins. We performed RNA pull-down assays in vitro with biotinylated SGO1-AS1, followed by SDS-PAGE electrophoresis, and an overtly differential band at approximately 60 kD in the sense lane was selected for mass spectrum analyses (Fig. 3a). Our results revealed several potential proteins that were pulled down with SGO1-AS1 RNA, and of these proteins, PTBP1 received the highest score (Additional file 1: Table S7). Biotin-labeled RNA pulldown followed by Western blotting analysis confirmed that PTBP1 and G3BP2 are SGO1-AS1-binding proteins (Fig. 3b). Furthermore, RIP followed by qRT-PCR assays showed that antibodies against either PTBP1 or G3BP2 could significantly enrich for SGO1-AS1 compared to the controls (Fig. 3c).

**Fig. 4:**
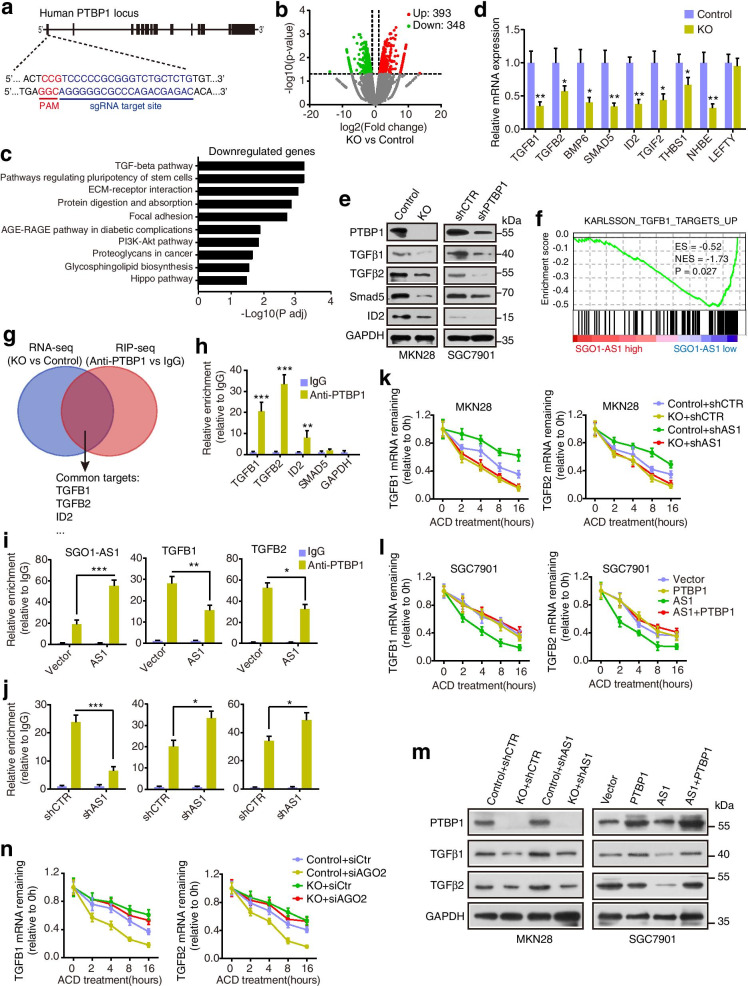
SGO1-AS1 promotes TGFB1/2 mRNA decay by interacting with PTBP1. **a.** PTBP1-knockout (KO) MKN28 cells were produced using the CRISPR/Cas9 system. Disruption of the PTBP1 locus. **b.** Volcano plots showing the differentially expressed genes in the PTBP1-KO vs. control cells. **c.** KEGG analysis of the downregulated genes in the PTBP1-KO vs. control cells. **d-e.** qRT-PCR and Western blot analysis were used to validate the expression of genes involved in the TGFβ pathway in the indicated cells. **f.** GSEA results plotted to illustrate the correlation between the expression of SGO1-AS1 and TGFβ target genes in the TCGA stomach adenocarcinoma RNA-seq dataset. **g.** Overlay of differentially expressed genes following the PTBP1 knockout and PTBP1-binding target mRNAs. **h.** PTBP1 RIP assay was performed to analyze the interactions between the PTBP1 protein and TGFB1/2 or ID2 mRNA in MKN28 cells. The relative fold enrichment of these mRNAs compared to IgG was determined by qRT-PCR. SMAD5 and GAPDH served as negative controls. **i-j.** The enrichment of SGO1-AS1 RNA and TGFB1/2 mRNA in PTBP1 immunoprecipitants was detected by a RIP-qPCR assay in SGC7901 cells with SGO1-AS1 overexpression **(i)** and MKN28 cells with SGO1-AS1 knockdown **(j)**, respectively. **k-l**. TGFB1/2 mRNA stability assessment in the indicated cells treated with actinomycin D (5 μg/mL) for 2, 4, 8 and 16 h. TGFB1/2 mRNA abundance relative to GAPDH quantified by qRT-PCR (*n* = 3 independent experiments). **m.** Western blotting analysis of the TGFβ1/2 and PTBP1 protein levels in the indicated cells. **n.** TGFB1/2 mRNA stability assessment in control and PTBP1-KO MKN28 cells transfected with siAGO2 or control siRNA. Error bars represent SDs. *P < 0.05, **P < 0.01, ***P < 0.001

PTBP1 is a member of the heterogeneous nuclear ribonucleoprotein (hnRNP) family and a critical regulator of mRNA splicing [15, 16], RNA stability [17], transportation [18], localization [19] and translation [20, 21]. PTBP1 has been shown to be involved in tumorigenesis, although its effects on malignancy appear to be cell-type dependent [16, 22, 23]. We focused on PTBP1 as an interacting partner of SGO1-AS1 for further investigation. To determine the region of SGO1-AS1 to which PTBP1 binds, we prepared a series of biotin-labeled SGO1-AS1 probes with deletion mutants and performed an in vivo RNA pull-down experiment. We found that the 1–415 nt fragment of SGO1-AS1 was sufficient to bind PTBP1 (Fig. 3d). Considering that PTBP1 binds pyrimidine-rich sequences (UCUUC), we identified the accurate binding site of PTBP1 in SGO1-AS1 (258–319 nt) via RNA pulldown assays (Fig. 3e). PTBP1 contains four RRM domains for binding RNA [24]. To investigate which domain of PTBP1 accounts for its interaction with SGO1-AS1, we performed RIP assays using a series of Flag-tagged PTBP1 deletion mutants and found that the RRM2 domain of PTBP1 had the strongest association with SGO1-AS1 (Fig. 3f). In addition, we observed that neither overexpression nor knockdown of SGO1-AS1 could influence the expression levels of PTBP1 (Fig. 4m).

### SGO1-AS1 regulates TGFB1/2 mRNA stability by interacting with PTBP1

PTBP1 is an RNA-binding protein (RBP) that plays a role in mRNA metabolism by binding target mRNAs [20]. We hypothesized that the association between SGO1-AS1 and PTBP1 may influence the effects of PTBP1 on its target mRNAs. We first evaluated the expression levels of PTBP1 in gastric cell lines and found it to be expressed at high levels in almost all GC cell lines (Additional file 1: Fig. S5a). Then, we generated a stable PTBP1-knockout MKN28 cell line using CRISPR-Cas9 technology (Fig. 4a, e) and performed an RNA-seq analysis. The comparison of the PTBP1-knockout and control cells revealed that 393 mRNAs were upregulated and 348 mRNAs were downregulated (Fig. 4b). The Kyoto Encyclopedia of Genes and Genomes (KEGG) analysis revealed that the downregulated genes were apparently enriched in TGFβ signaling and pathways regulating stem cells, the extracellular matrix (ECM) and focal adhesion (Fig. 4c). The downregulated genes in the PTBP1-knockout MKN28 cells involved in the TGFβ pathway were further validated at the mRNA and protein levels (Fig. 4d, e). A similar result was observed in the SGC7901 cells transfected with PTBP1 shRNA (Additional file 1: Fig. S5b). Interestingly, based on the SGO1-AS1 levels, we performed a gene set enrichment analysis (GSEA) using The Cancer Genome Atlas (TCGA: https://cancergenome.nih.gov) stomach adenocarcinoma RNA-seq dataset and demonstrated that TGFβ1 target genes were significantly enriched in GC patients with low SGO1-AS1 expression (Fig. 4f), implying a role of this lncRNA in regulating TGFβ signaling.

Given that PTBP1 is an RNA binding protein [20], we performed RIP-seq experiments using an anti-PTBP1 antibody or rabbit immunoglobulin G to reveal the PTBP1-bound RNAs. We identified 5186 transcripts potentially bound by PTBP1 and 173 transcripts that overlapped between the PTBP1-bound mRNAs and differentially expressed genes following PTBP1 knockout; three of these transcripts (TGFB1, TGFB2 and ID2) were related to the TGFβ pathway (Fig. 4g). The interaction between PTBP1 and TGFB1, TGFB2 or ID2 mRNA was further confirmed by RIP followed by qPCR (Fig. 4h and Additional file 1: Fig. S5c). Importantly, SGO1-AS1 overexpression resulted in a large increase in the association between SGO1-AS1 and PTBP1, while the TGFB1/2 mRNA binding of PTBP1 was markedly decreased in the SGC7901 cells (Fig. 4i). In contrast, the association between TGFB1/2 mRNA and PTBP1 was increased following the knockdown of SGO1-AS1 in the MKN28 cells (Fig. 4j). However, the regulation of SGO1-AS1 expression did not influence the association between the PTBP1 protein and ID2 mRNA (Additional file 1: Fig. S5d). Furthermore, we found that the TGFB1/2 mRNA mainly bound the RRM2 domain of PTBP1 (Additional file 1: Fig. S5e), which is the region bound by SGO1-AS1 (Fig. 3f). Altogether, these results suggest that SGO1-AS1 competes with TGFB1/2 mRNA to bind PTBP1.

Since our above results indicate that depletion of PTBP1 reduces the TGFB mRNA levels, we investigated whether PTBP1 and SGO1-AS1 may influence TGFB mRNA stability. Actinomycin D (ACD) was used to block de novo transcription in PTBP1-knockout and control cells infected with SGO1-AS1 shRNA. As expected, the depletion of PTBP1 increased TGFB mRNA degradation following ACD treatment in MKN28 cells. However, silencing SGO1-AS1 stabilized TGFB mRNAs but had no significant effect on TGFB mRNA stability under conditions in which PTBP1 was depleted (Fig. 4k). Furthermore, the forced expression of SGO1-AS1 facilitated TGFB1/2 mRNA degradation, and the co-overexpression of PTBP1 abrogated this effect, although the overexpression of PTBP1 alone failed to influence TGFB mRNA stability (Fig. 4l), indicating that PTBP1 is necessary for the SGO1-AS1-mediated TGFB mRNA decay. Consistently, knockdown of SGO1-AS1 increased the TGFβ1/2 protein level, which was offset following PTBP1 knockout (Fig. 4m). Conversely, SGO1-AS1 overexpression remarkably downregulated TGFβ protein expression, and this downregulation was reversed by the coexpression of PTBP1 (Fig. 4m). Moreover, the xenograft tumors generated by SGO1-AS1-overexpressing SGC7901 cells featured lower TGFB1/2 expression than the control tumors (Additional file 1: Fig. S5f, g). Overall, these results demonstrate that SGO1-AS1 inhibits TGFB1/2 mRNA expression via a PTBP1-mediated mechanism.

Next, we explored the mechanism by which PTBP1 promotes the stability of TGFB1/2 mRNA. Numerous reports indicate that the RNA-induced silencing complex (RISC) mediates mRNA decay [25, 26], and we hypothesized that this mechanism may account for the PTBP1-mediated TGFB stability. To address this, we knocked down AGO2, a major element of RISC, with siRNAs and found that knockdown of AGO2 increased TGFB1/2 expression levels in PTBP1-KO cells (Additional file 1: Fig. S5h). Moreover, the TGFB mRNA degradation was decreased in PTBP1-KO cells transfected with siAGO2 compared with the control (Fig. 4n). We next determined whether AGO2 complexes with TGFB1/2 mRNA. RIP assays showed that AGO2 could be significantly enriched in TGFB mRNA (Additional file 1: Fig. S5i). Importantly, depletion of PTBP1 significantly enhanced but silencing of SGO1-AS1 reduced the enrichment of AGO2 in TGFB1/2 mRNA (Additional file 1: Fig. S5h). Together, these results indicate that PTBP1 prevents AGO2 recruitment of TGFB mRNA to form RISC system, causing a decrease in decay of TGFB mRNA.

### SGO1-AS1 impedes TGFβ signaling, the EMT and stemness

**Fig. 5:**
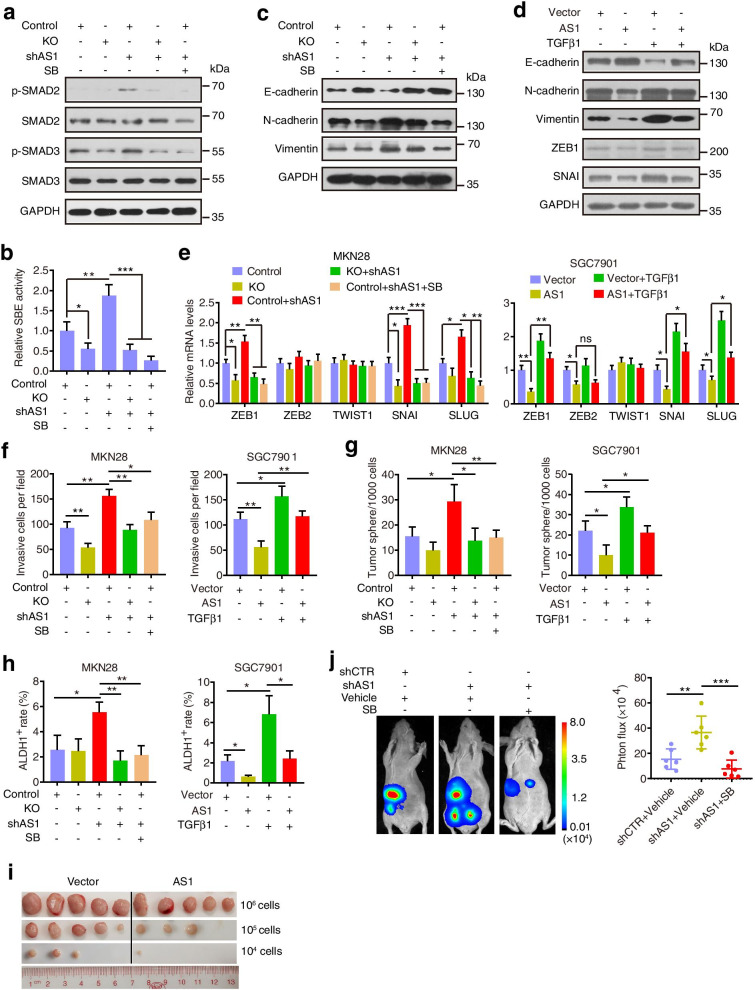
SGO1-AS1 impedes TGFβ signaling, the EMT and stemness. **a-b.** SGO1-AS1 knockdown increased SMAD2/3 phosphorylation and SBE4 transcriptional activity, and this effect was reversed by PTBP1 knockout or SB431542 (10 μM, 24 h) as shown by Western blot **(a)** and luciferase reporter assays of the SBE4 promoter **(b)**. **c-d.** EMT markers were measured in the indicated cells by a Western blot analysis. **e.** qRT-PCR analysis of EMT-TF expression in the indicated cells. **f-h.** Cell invasion ability **(f)**, tumorsphere formation efficiency **(g)** and rates of ALDH1-positive cells **(h)** in the indicated cells. **i.** Limiting dilution xenograft formation of SGC7901 cells stably expressing SGO1-AS1 or vector (n = 5 mice per group)**. j.** MKN28 cells with SGO1-AS1 knockdown or control were orthotopically injected into the stomach of nude mice, and the mice were treated with PBS or SB431542 (20 mg/kg body weight, i.p.) three times per week for 3 weeks (*n* = 6 mice per group). Tumor progression was monitored by luciferase signal intensity using an In Vivo Imaging System. Error bars indicate SDs. *P < 0.05, **P < 0.01, ***P < 0.001

As SGO1-AS1 reduces TGFβ production, we evaluated whether SGO1-AS1 may affect TGFβ downstream signaling. As expected, silencing SGO1-AS1 enhanced the phosphorylation of SMADs and induced the transcriptional activity of SBE4 (SMAD-binding element) in control cells but showed little effect in the PTBP1-deficient cells, and the activation of TGFβ signaling by SGO1-AS1 knockdown was reversed by the treatment with the TGFβ type I receptor (TβRI) inhibitor SB431542 (Fig. 5a, b). These results indicate that SGO1-AS1 acts as a potent antagonist of TGFβ/SMAD signaling in a PTBP1-dependent manner.

TGFβ is a major inducer of the EMT and stimulates stemness, invasion and metastasis in cancer cells [27, 28]. Therefore, we examined whether silencing SGO1-AS1 could promote the ability of GC cells to undergo the EMT. As shown in Fig. 5c and Additional file 1: Fig. S6a, knockdown of SGO1-AS1 in MKN28 cells reduced the epithelial features but increased the mesenchymal features as evidenced by the reduced expression of an epithelial marker (E-cadherin) but increased levels of mesenchymal markers (Vimentin and N-cadherin) and the elongation of cell bodies. We also examined the levels of the EMT-inducing transcription factors (EMT-TFs) ZEB1, ZEB2, TWIST1, SNAI and SLUG and showed that the expression levels of ZEB1, SNAI and SLUG were increased upon SGO1-AS1 knockdown (Fig. 5e). Moreover, the SGO1-AS1 knockdown-induced mesenchymal-like features were reversed by PTBP1 knockout or SB431542 treatment (Fig. 5c, e and Additional file 1: Fig. S6a). In contrast, SGO1-AS1 overexpression in SGC7901 cells dampened the EMT process, which was rescued by the treatment with the recombinant TGFβ1 protein (Fig. 5d, e and Additional file 1: Fig. S6a). Consistently, the invasive activity was significantly decreased in the SGO1-AS1-deleted MKN28 cells upon PTBP1 loss or SB431542 treatment but was rescued in the SGO1-AS1-overexpressing SGC7901 cells with the addition of recombinant TGFβ1 (Fig. 5f and Additional file 1: Fig. S6b). To confirm the inhibitory effects of SGO1-AS1 on metastatic activity through TGFβ signaling in vivo, we orthotopically implanted SGO1-AS1 knockdown cells into nude mice and treated them with SB431542 (three times per week). Consistent with the overexpression study (Fig. 2g), the metastasis signals found in the SGO1-AS1 knockdown group were higher than the signals in the control group, while the SB431542 treatment exhibited a significant reduction in metastases in the mice harboring SGO1-AS1 knockdown cells (Fig. 5j).

Then, we examined the effects of SGO1-AS1 on stemness. SGO1-AS1 knockdown increased tumor spheroid formation and the ALDH1+ population, whereas loss of PTBP1 or treatment with SB431542 significantly abolished these effects (Fig. 5g, h and Additional file 1: Fig. S6c). In contrast, SGO1-AS1 overexpression reduced the stemness features in SGC7901 cells, and the addition of TGFβ1 induced stemness features in the SGO1-AS1-overexpressing cells. Moreover, the in vivo limiting dilution tumorigenicity assays showed that the SGO1-AS1 overexpression also inhibited the tumorigenic potential of the SGC7901 cells in the nude mice (Fig. 5i). Collectively, these data indicate that SGO1-AS1 attenuates the EMT, stemness and metastasis via PTBP1-mediated TGFβ signaling.

### SGO1-AS1 reduces TGFβ autocrine signaling

**Fig. 6:**
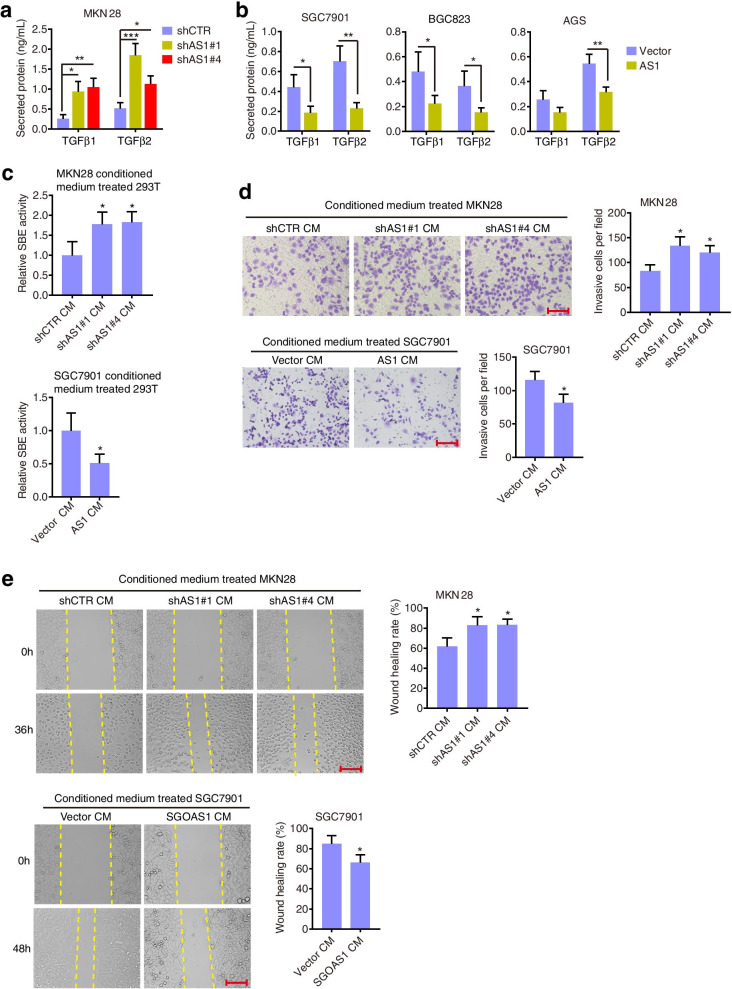
SGO1-AS1 decreases TGFβ autocrine signaling. **a-b.** ELISA analysis of the TGFβ1/2 protein levels in conditioned medium from cells with SGO1-AS1 knockdown **(a)** or overexpression **(b)**. **c.** Conditioned medium from SGO1-AS1-silenced MKN28 cells enhanced SBE4 promoter luciferase activity in HEK293T cells. In contrast, SBE4 transcription in HEK293T cells treated with conditioned medium collected from the SGO1-AS1-overexpressing SGC7901 cells was downregulated compared to that in the cells treated with medium collected from the control cells. **d.** The cell invasion ability of MKN28 cells increased when treated with conditioned medium from SGO1-AS1-silenced MKN28 cells, but that of SGC7901 cells was decreased following the treatment with conditioned medium from SGO1-AS1-overexpressing SGC7901 cells. **e.** The migratory ability of MKN28 cells was increased following the treatment with conditioned medium from SGO1-AS1-silenced MKN28 cells, while that of SGC7901 cells was decreased following the treatment with conditioned medium from SGO1-AS1-overexpressing SGC7901 cells. Scale bar, 150 μm. All error bars indicate SDs. *P < 0.05, **P < 0.01, ***P < 0.001

Because our results showed that SGO1-AS1 reduced TGFβ expression and its downstream signaling, we reasoned that SGO1-AS1 might influence the secretion of these cytokines to interrupt the tumor microenvironment. Indeed, SGO1-AS1 knockdown induced, but SGO1-AS1 overexpression inhibited, the secretion of these cytokines as observed in enzyme-linked immunosorbent assays (ELISAs) (Fig. 6a, b). To confirm that SGO1-AS1 modulates TGFβ autocrine signaling, we collected conditioned medium from SGO1-AS1-knockdown MKN28 cells or SGO1-AS1-overexpressing SGC7901 cells, transferred the conditioned medium to HEK293T cells and performed SBE4 transcription assays. The results showed that the conditioned medium collected from the SGO1-AS1-silenced cells activated SBE4 transcription (Fig. 6c). In contrast, SBE4 transcription in HEK293T cells treated with conditioned medium collected from SGO1-AS1-overexpressing cells was downregulated compared to that in the control cells. Next, we explored the invasive phenotype of GC cells incubated with conditioned medium. As expected, MKN28 cells incubated with conditioned medium collected from SGO1-AS1-silenced MKN28 cells exhibited higher migration and invasion capacities than the cells incubated with conditioned medium collected from control cells, whereas SGC7901 cells incubated with conditioned medium collected from SGO1-AS1-overexpressing SGC7901 cells had reduced migration and invasion abilities compared to the cells incubated with conditioned medium collected from control SGC7901 cells (Fig. 6d, e). These data indicate that SGO1-AS1 reduces the autocrine activity of TGFβ cytokines.

### TGFβ represses SGO1-AS1 transcription via ZEB1

**Fig. 7:**
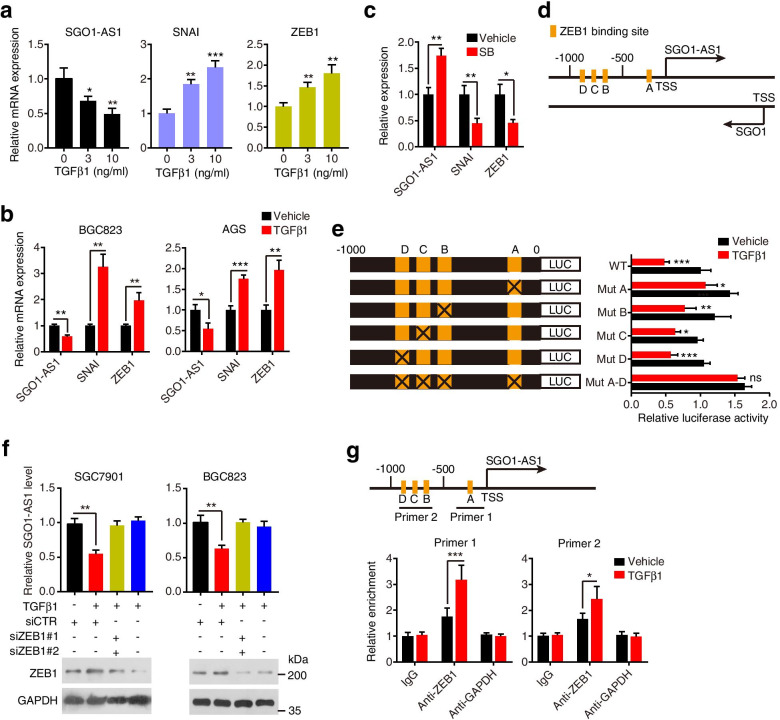
TGFβ downregulates SGO1-AS1 transcription via ZEB1. **a.** qRT-PCR analyses of the SGO1-AS1, SNAI and ZEB1 levels in SGC7901 cells incubated with TGFβ1 (3 ng/mL or 10 ng/mL) for 24 h. **b.** Expression levels of SGO1-AS1, SNAI and ZEB1 in BGC823 and AGS cells stimulated with 10 ng/mL TGFβ1 for 24 h. *P < 0.05, **P < 0.01 and ***P < 0.001 compared to those without TGFβ1 stimulation. **c.** SGO1-AS1, SNAI and ZEB1 levels in SGC7901 cells incubated with SB431542 (10 μM) for 24 h. *P < 0.05, **P < 0.01. **d.** A schematic diagram illustrating the four putative ZEB1 binding sites (Sites A, B, C and D) in the SGO1-AS1 promoter. **e.** Luciferase reporter assays of the SGO1-AS1 promoter region containing either wild-type (WT) or mutated (Mut A, Mut B, Mut C, Mut D, or Mut A-D) ZEB1 binding sites without or with TGFβ1 exposure. *P < 0.05, **P < 0.01, ***P < 0.001 and ns, not significant compared to without the TGFβ1 stimulation. **f.** SGO1-AS1 expression was examined by a qRT-PCR analysis in TGFβ1-treated SGC7901 and BGC823 cells transfected with ZEB1 siRNAs. Western blot analysis was performed to assess the inhibition efficiency in the same cells (right). **P < 0.01. **g.** Upper: Putative ZEB1-binding sites on the SGO1-AS1 promoter region and design-indicated primers. Lower: ChIP analysis of ZEB1 enrichment on the SGO1-AS1 promoter in SGC7901 cells treated with TGFβ1. IgG and anti-GAPDH antibodies were used as controls. *P < 0.05, ***P < 0.001. In all cases, error bars indicateSDs from three independent experiments

Having found that SGO1-AS1 antagonizes TGFβ signaling by promoting TGFB mRNA degradation, we investigated the response of SGO1-AS1 to TGFβ. We treated SGC7901 cells with TGFβ1 and found that SGO1-AS1 expression was inhibited, but well-known TGFβ targets (SNAI and ZEB1) [29, 30] were induced in a dose-dependent manner (Fig. 7a). SGO1-AS1 downregulation by the TGFβ treatment was confirmed in two other GC cell lines (Fig. 7b). Then, we exposed the cells to the TGFβRI inhibitor SB431542 and found that the SB431542 treatment induced SGO1-AS1 expression, which was accompanied by the downregulation of SNAI and ZEB1 (Fig. 7c). We carried out a bioinformatics analysis using JASPAR (http://jaspar.genereg.net/) to identify potential regulatory transcription factors. The bioinformatics analysis revealed four ZEB1 binding motifs at − 222 to − 214 (site A), − 731 to − 721 (site B), − 822 to − 814 (site C) and − 947 to − 940 (site D) inside the SGO1-AS1 promoter (Fig. 7d). ZEB1 is a downstream target gene of TGFβ and has been reported to be a master regulator of the EMT and cancer metastasis [31]. We evaluated whether the downregulation effects of TGFβ on SGO1-AS1 occur via ZEB1. The luciferase reporter assay showed that the luciferase activity of the reporter constructs containing 1 kb of the wild-type SGO1-AS1 promoter was decreased by approximately 2-fold in the TGFβ-treated cells relative to that in the control cells, and the ZEB1 binding site mutation on the SGO1-AS1 promoter abolished this effect (Fig. 7e). Then, we knocked down ZEB1 in GC cells treated with TGFβ1 to study its effects on the SGO1-AS1 levels and found that depletion of ZEB1 completely reversed the repressive effects of TGFβ1 on SGO1-AS1 expression (Fig. 7f). Using ChIP, we discovered significant enrichment in the binding of ZEB1 to the promoter region of SGO1-AS1 in the cells treated with TGFβ1 relative to the control cells (Fig. 7g). Taken together, our data indicate that TGFβ downregulates SGO1-AS1 by inducing ZEB1.

### SGO1-AS1 downregulation is correlated with high expression of TGFB1/2 and ZEB1 in gastric carcinoma specimens

**Fig. 8:**
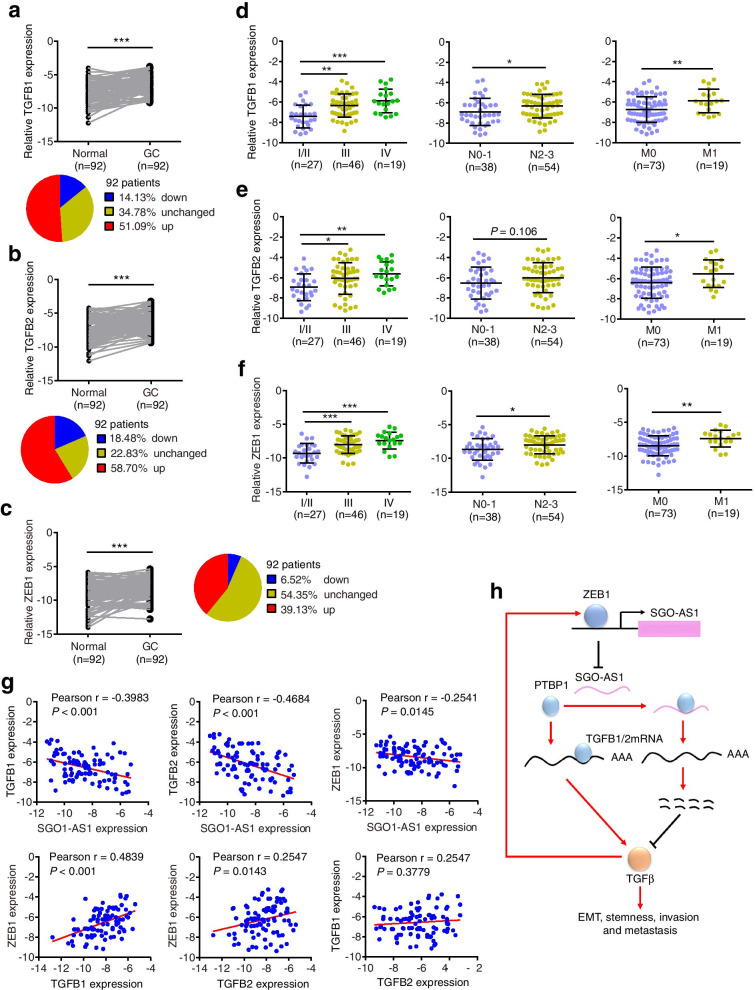
Expression levels of TGFB1/2 and ZEB1 and their correlations with SGO1-AS1 expression in GC tissues. **a-c.** Relative expression levels of TGFB1, TGFB2 and ZEB1 in 92 paired GC and normal tissues from Cohort 2 were quantified by qRT-PCR. The pie charts show the proportions of samples in the downregulation (blue), upregulation (red) and no change (yellow) categories. **d-f.** The expression levels of TGFB1, TGFB2 and ZEB1 in GCs according to their clinical stage and status of lymph node or distant metastasis. **g.** SGO1-AS1 expression was inversely correlated with TGFB1, TGFB2 and ZEB1 expression, while ZEB1 expression was positively correlated with TGFB1/2 expression in the GC specimens. Error bars indicate SDs. *P < 0.05, **P < 0.01, ***P < 0.001. **h.** Schematic illustration of the TGFβ/ZEB1/SGO1-AS1 signaling pathway. TGFβ downregulates SGO1-AS1 by inducing ZEB1. SGO1-AS1 inactivates TGFβ signaling by promoting TGFB mRNA decay

We sought to determine whether our findings could be extended to patients with gastric carcinoma. The expression levels of TGFB1, TGFB2, ZEB1 and PTBP1 were detected by qRT-PCR in 92 pairs of gastric carcinoma specimens and adjacent normal tissues (Cohort 2). The expression levels of these four genes were significantly increased in the tumors compared to those in the normal tissues, and 51.1% (47/92), 58.7% (54/92) 39.1% (36/92), and 52.2% (48/92) of the GC samples showed more than a 2-fold upregulation of TGFB1, TGFB2, ZEB1 and PTBP1, respectively (Fig. 8a-c and Additional file 1: Fig. S7a). Moreover, high levels of TGFB1/2 and ZEB1 were significantly associated with lymphatic invasion and advanced tumor stage (Fig. 8d-f). GCs with metastasis expressed higher levels of TGFB1/2, ZEB1 and PTBP1 than those without metastasis (Fig. 8d-f and Additional file 1: Fig. S7b). Importantly, TGFB1/2 and ZEB1 were inversely correlated with SGO1-AS1, whereas TGFB1/2 was positively associated with ZEB1 in the GC tissues (Fig. 8g), further confirming the SGO1-AS1-TGFB-ZEB1 regulatory axis in GC. In addition, there was no significant correlation between PTBP1 and SGO1-AS1 expression or between TGFB1 and TGFB2 expression (Fig. 8g and Additional file 1: Fig. S7c). We also observed that PTBP1 was significantly associated with TGFB1 expression (Additional file 1: Fig. S7c).

## Discussion

Most patients with GC die from metastatic disease, but knowledge regarding the mechanisms of metastasis in gastric tumors is limited [2]. In this study, we identified a metastasis-suppressive lncRNA, i.e., SGO1-AS1, which is decreased in progressed gastric cancer and inversely correlated with gastric tumor metastasis. We further revealed that SGO1-AS1 interacts with the protein PTBP1, and their interaction competitively reduces TGFB1/2 mRNA binding to PTBP1. In turn, the decreased binding of TGFB1/2 mRNA to PTBP1 leads to a reduction in TGFB1/2 mRNA stability and reduced TGFβ production, thus preventing the EMT and metastasis. In addition, TGFβ represses SGO1-AS1 transcription by inducing ZEB1. Thus, SGO1-AS1 and TGFβ form a double-negative feedback loop via ZEB1 to regulate the EMT and metastasis (Fig. 8h).

LncRNAs often exert their effects through the proteins with which they interact [32]. Here, we identified PTBP1 as an SGO1-AS1-interacting protein. PTBP1 has been shown to be involved in tumorigenesis by regulating alternative splicing [33, 34], controlling mRNA stability [17, 35] and determining mRNA localization [19]. For example, PTBP1 enhances the PKM2 isoform and reduces the PKM1 isoform by controlling PKM alternative splicing, which promotes aerobic glycolysis and provides a selective advantage for tumor formation [16, 36]. PTBP1 mediates MCL1 mRNA stability and regulates cellular apoptosis induced by antitubulin chemotherapeutics [23]. Notably, several lncRNAs have been reported to be associated with PTBP1 [17, 37, 38]. The hypoxia-induced lncRNA LUCAT1 interacts with PTBP1 in CRC cells, facilitating the association between a set of DNA damage-related genes and PTBP1 and resulting in altered alternative splicing of these genes, thereby conferring resistance to chemotherapeutic drugs in CRC cells [39]. The lncRNA MEG3 can recruit PTBP1 to regulate small heterodimer partner mRNA stability and cholestatic liver injury [17]. Recruiting PTBP1 to target mRNAs appears to be a common mechanism among these lncRNAs. However, we found that the interaction between SGO1-AS1 and PTBP1 reduces the enrichment of this protein in TGFB1/2 mRNA to facilitate their decay. In addition to PTBP1, it is possible that SGO1-AS1 might bind other proteins, such as G3BP2, to regulate GC metastasis as G3BP2 was found to be an SGO1-AS1-interacting protein in the mass spectrum analyses and verification analyses in our study. The role of this and other proteins bound by SGO1-AS1 in gastric carcinoma deserves further investigation.

Identifying TGFβ-induced ZEB1 as a potent transcriptional repressor of SGO1-AS1 is another important finding of this study. Here, we demonstrate that a reciprocal negative feedback loop exists between SGO1-AS1 and TGFβ/ZEB1. Although the double positive feedback loop between TGFβ and lncRNA is well documented [40–42], to the best of our knowledge, the reciprocal repressive loop between TGFβ and lncRNA has rarely been observed. Our current study provides evidence of a reciprocal repressive loop between TGFβ and the lncRNA SGO1-AS1 in GC metastasis. We show that ZEB1 induced by TGFβ transcriptionally inhibits SGO1-AS1 expression; in turn, SGO1-AS1 inhibits TGFβ expression by reducing TGFB mRNA stability, which mediates the reciprocal repressive loop between TGFβ/ZEB1 and SGO1-AS1 in GC.

TGFβ signaling is highly conserved in multicellular organisms and is involved in multiple cellular processes, such as cell growth, stemness, migration, invasion, the EMT, ECM, remodeling and immune regulation [43]. The activation of canonical TGFβ signaling is caused by the binding of TGFβ ligands (TGFβ1, TGFβ2 and TGFβ3) to heteromeric TGFβ type I and II receptors, which phosphorylate SMAD2 and SMAD3, resulting in complex formation with SMAD4 and nuclear translocation to regulate target gene transcription [44]. TGFβ plays a critical role in tumorigenesis and tumor progression in a complex and pleiotropic manner; in early tumor initiation, it plays a tumor-suppressive role by inhibiting cell proliferation and stimulating apoptosis; however, in advanced tumors, it promotes tumor progression by inducing the EMT, which is correlated with increased invasiveness, metastasis and chemoresistance in tumor cells [45, 46]. Because of its role in advanced tumors, TGFβ is considered a therapeutic target. Several strategies have been proposed to inhibit TGFβ signaling to combat malignant tumors (e.g., small-molecule inhibitors of receptor kinases, TGFβ neutralizing antibodies and antisense compounds) [47]. Our finding that SGO1-AS1 and TGFβ/ZEB1 form a double-negative feedback loop hints at the possibility of new therapeutic approaches to block the TGFβ signal by introducing SGO1-AS1 or using the interference of ZEB1, although this possibility remains to be confirmed by future studies.

## Conclusions

Our study identified a metastasis-suppressive lncRNA that functions as an endogenous inhibitor of the TGFβ pathway and suppresses GC metastasis and progression. Our data further highlight the importance of the double-negative feedback loop between SGO1-AS1 and TGFβ/ZEB1 in GC metastasis. These findings provide novel information for understanding the mechanisms underlying the pathogenesis in GC metastasis and new insight into the potential use of SGO1-AS1-TGFβ-ZEB1 for the development of new treatment strategies for GC.

## Supplementary Information

**Additional file 1: Fig. S1** SGO1-AS1 was downregulated in GC tissues. Expression levels of SGO1-AS1 and 12 other lncRNAs validated by qRT-PCR in 18 paired gastric cancer tissues and adjacent normal tissues (Cohort 1). The results are expressed as -∆Ct. **P < 0.05, **P < 0.01, ***P < 0.001. **Fig. S2** Characterization of human SGO1-AS1 as a long noncoding RNA. a. Schematic diagram of the genomic locus and isoforms of SGO1-AS1 in the UCSC Genome Browser (http://genome.ucsc.edu/). b. Identification of full-length SGO1-AS1 by 5′ and 3′ RACE. Left: Representative images of the PCR products from 5′ RACE and 3′ RACE. Right: Nucleotide sequence of full-length human SGO1-AS1. c. Coding potential of SGO1-AS1 as predicted by the CPAT and CPC tools. The lncRNA MALAT1 and the protein-coding genes GAPDH and ACTB are also shown. d. Relative distribution of SGO1-AS1 in gastric cell lines as determined by RT-PCR. SGO1-AS1 was mainly expressed in the cytoplasm of epithelial cells. **Fig. S3** SGO1-AS1 inhibits GC cell migration and growth. a. qRT-PCR showed the relative levels of SGO1-AS1 expression in human gastric cancer cell lines compared to those in the immortalized human gastric epithelial cell line GES1. b. qRT-PCR analysis was used to confirm the level of SGO1-AS1 in cells with stable overexpression of SGO1-AS1. c. qRT-PCR analysis was performed to assess the inhibition efficiency in MKN28 cells infected with SGO1-AS1 shRNA lentiviruses targeting different regions of SGO1-AS1. d. Cell migration in BGC823 cells stably expressing SGO1-AS1 and control cells was examined by a wound healing assay. Scale bar, 150 μm. e. Cell proliferation assay in SGC7901 and BGC823 cells stably expressing SGO1-AS1, MKN28 cells stably silencing SGO1-AS1 and the respective control cells. f-g. A soft agar colony formation assay was carried out in the indicated cells. Scale bars, 100 μm. h. Overexpression of SGO1-AS1 inhibits GC tumor growth in a nude mouse model. SGC-7901 cells with stable overexpression of SGO1-AS1 or the control were inoculated subcutaneously into nude mice (*n* = 6 mice/group). The mice were sacrificed at 28 days postinoculation, and the tumors were weighed. Error bars indicate SD. *P < 0.05, **P < 0.01, ***P < 0.001. **Fig. S4** Effects of SGO1-AS1 on the expression of the sense gene SGO1. a-b. qRT-PCR and Western blotting analyses of the SGO1 levels in SGC7901 and BGC823 cells stably expressing SGO1-AS1, MKN-28 cells stably silencing SGO1-AS1 and the related control cells. Error bars indicate SDs from three independent experiments. *P < 0.05, **P < 0.01. **Fig. S5** SGO1-AS1 reduces TGFB1/2 mRNA stability. a. Western blot analysis was used to evaluate PTBP1 expression in human gastric cancer cell lines and normalgastric epithelial cell line. b. qRT-PCR analysis was performed to examine the expression levels of genes related to the TGFβ pathway in SGC7901 cells transfected with PTBP1 shRNA or the control. c. PTBP1 RIP assay was performed to analyze the interactions between PTBP1 and TGFB1/2 or ID3 in SGC7901 cells. The relative fold enrichment of these mRNAs compared to IgG was determined by qRT-PCR. SMAD5 and GAPDH were used as negative controls. d. RIP-qPCR assay was used to evaluate the interaction between PTBP1 and ID2 mRNA in MKN28 cells with SGO1-AS1 knockdown or SGC7901 cells with SGO1-AS1 overexpression. e. Deletion mapping was performed to determine the binding domain of PTBP1 to TGFB1/2 mRNA using full-length or truncated PTBP1. f-g. mRNA (f) and protein (g) expression levels of TGFβ1/2 in xenograft tumor tissues recovered from nude mice. #1–3 denote individual tumors grown in different mice. h. Western blot analysis of AGO2, TGFβ1 and TGFβ2 in control and PTBP1-KO MKN28 cells transfected with siAGO2 or control siRNA. i. RIP-qPCR assay of the interaction between the AGO2 protein and TGFB1/2 mRNA in MKN28 cells with PTBP1 knockout or SGO1-AS1 knockdown. Error bars are SDs from three independent experiments. *P < 0.05, **P < 0.01, ***P < 0.001 and ns, not significant. **Fig. S6** SGO1-AS1 changes cell morphology and suppresses cell invasion and stemness. a-c. Representative phase-contract (a), cell invasion (b) and tumor spheroid images (c) of the indicated cells are shown. a, Scale bars,100 μm; b-c, Scale bars, 150 μm. **Fig. S7** Expression level of PTBP1 and its correlation with SGO1-AS1, TGFB1/2 and ZEB1 expression in GC tissues. a. Relative expression levels of PTBP1 in 92 paired GC and normal tissues from Cohort 2 were quantified by qRT-PCR. The pie chart shows the proportions of samples in the downregulation (blue), upregulation (red) and no change (yellow) categories. b. The expression level of PTBP1 in GCs according to their clinical stage and status of lymph node or distant metastasis. c. Correlation between PTBP1 expression and SGO-AS1, TGFB1, TGFB2 and ZEB1 expression in GC specimens. a-c, Error bars indicate SDs. *P < 0.05, **P < 0.01, ***P < 0.001 and ns, not significant. **Table S1.** Clinicopathological characteristics of Cohort 2 samples. **Table S2.** Clinicopathological characteristics of Cohort 3 samples. **Table S3.** Primers used for quantitative PCR. **Table S4.** Primers for r*apid amplification of cDNA* ends analysis. **Table S5.** Correlation between clinicopathological parameters and SGO1-AS1 levels in 95 cases of GC tissues (Cohort 3). **Table S6.** Univariate and multivariate analyses of factors associated with overall survival. **Table S7.** Mass spectrometry protein identification results for biotinylated SGO1-AS1 RNA pull down.

## Data Availability

The microarray data have been deposited in the GEO data base under accession numbers GSE157289, GSE157582 and GSE157941.

## Abbreviations

lncRNAs: Long noncoding RNAs
GC: Gastric carcinoma
qRT-PCR: Quantitative reverse transcription polymerase chain reaction
ISH: In situ hybridization
EMT: Epithelial-to-mesenchymal transition
GEO: Gene expression omnibus
qPCR: Quantitative polymerase chain reaction
RACE: Rapid amplification of cDNA ends
DMEM: Dulbecco’s modified eagle’s medium
shRNA: Short hairpin RNA
sgRNA: Small guide RNA
SDS-PAGE: Sodium dodecyl sulfate-polyacrylamide gel electrophoresis
RIP: RNA immunoprecipitation
RNA-seq: RNA sequencing
ChIP: Chromatin immunoprecipitation
NC: Normal control
SD: Standard deviation
CPAT: Coding-potential assessment tool
CPC: Coding potential calculator
hnRNP: Heterogeneous nuclear ribonucleoprotein
RBP: RNA-binding protein
KO: Knockout
KEGG: Kyoto Encyclopedia of Genes and Genomes
ECM: Extracellular matrix
GSEA: Gene set enrichment analysis
TCGA: The cancer genome atlas
ACD: Actinomycin D
TβRI: TGFβ type I receptor
EMT-TFs: EMT-inducing transcription factors
ELISA: Enzyme linked immunosorbent assay
WT: Wild-type
