# Supplementary material for: Long noncoding RNA SGO1-AS1 inactivates TGFβ signaling by facilitating TGFB1/2 mRNA decay and inhibits gastric carcinoma metastasis
Source: J Exp Clin Cancer Res. 2021 Oct 28;40:342. doi: 10.1186/s13046-021-02140-0 (PMC8555099; doi:10.1186/s13046-021-02140-0)
Supplement: Supplementary file 1 — Additional file 1: Fig. S1 SGO1-AS1 was downregulated in GC tissues. Expression levels of SGO1-AS1 and 12 other lncRNAs validated by qRT-PCR in 18 paired gastric cancer tissues and adjacent normal tissues (Cohort 1). The results are expressed as -∆Ct. **P < 0.05, **P < 0.01, ***P < 0.001. Fig. S2 Characterization of human SGO1-AS1 as a long noncoding RNA. a. Schematic diagram of the genomic locus and isoforms of SGO1-AS1 in the UCSC Genome Browser (http://genome.ucsc.edu/). b. Identification of full-length SGO1-AS1 by 5′ and 3′ RACE. Left: Representative images of the PCR products from 5′ RACE and 3′ RACE. Right: Nucleotide sequence of full-length human SGO1-AS1. c. Coding potential of SGO1-AS1 as predicted by the CPAT and CPC tools. The lncRNA MALAT1 and the protein-coding genes GAPDH and ACTB are also shown. d. Relative distribution of SGO1-AS1 in gastric cell lines as determined by RT-PCR. SGO1-AS1 was mainly expressed in the cytoplasm of epithelial cells. Fig. S3 SGO1-AS1 inhibits GC cell migration and growth. a. qRT-PCR showed the relative levels of SGO1-AS1 expression in human gastric cancer cell lines compared to those in the immortalized human gastric epithelial cell line GES1. b. qRT-PCR analysis was used to confirm the level of SGO1-AS1 in cells with stable overexpression of SGO1-AS1. c. qRT-PCR analysis was performed to assess the inhibition efficiency in MKN28 cells infected with SGO1-AS1 shRNA lentiviruses targeting different regions of SGO1-AS1. d. Cell migration in BGC823 cells stably expressing SGO1-AS1 and control cells was examined by a wound healing assay. Scale bar, 150 μm. e. Cell proliferation assay in SGC7901 and BGC823 cells stably expressing SGO1-AS1, MKN28 cells stably silencing SGO1-AS1 and the respective control cells. f-g. A soft agar colony formation assay was carried out in the indicated cells. Scale bars, 100 μm. h. Overexpression of SGO1-AS1 inhibits GC tumor growth in a nude mouse model. SGC-7901 cells with stable overexpression of [file 13046_2021_2140_MOESM1_ESM.docx]

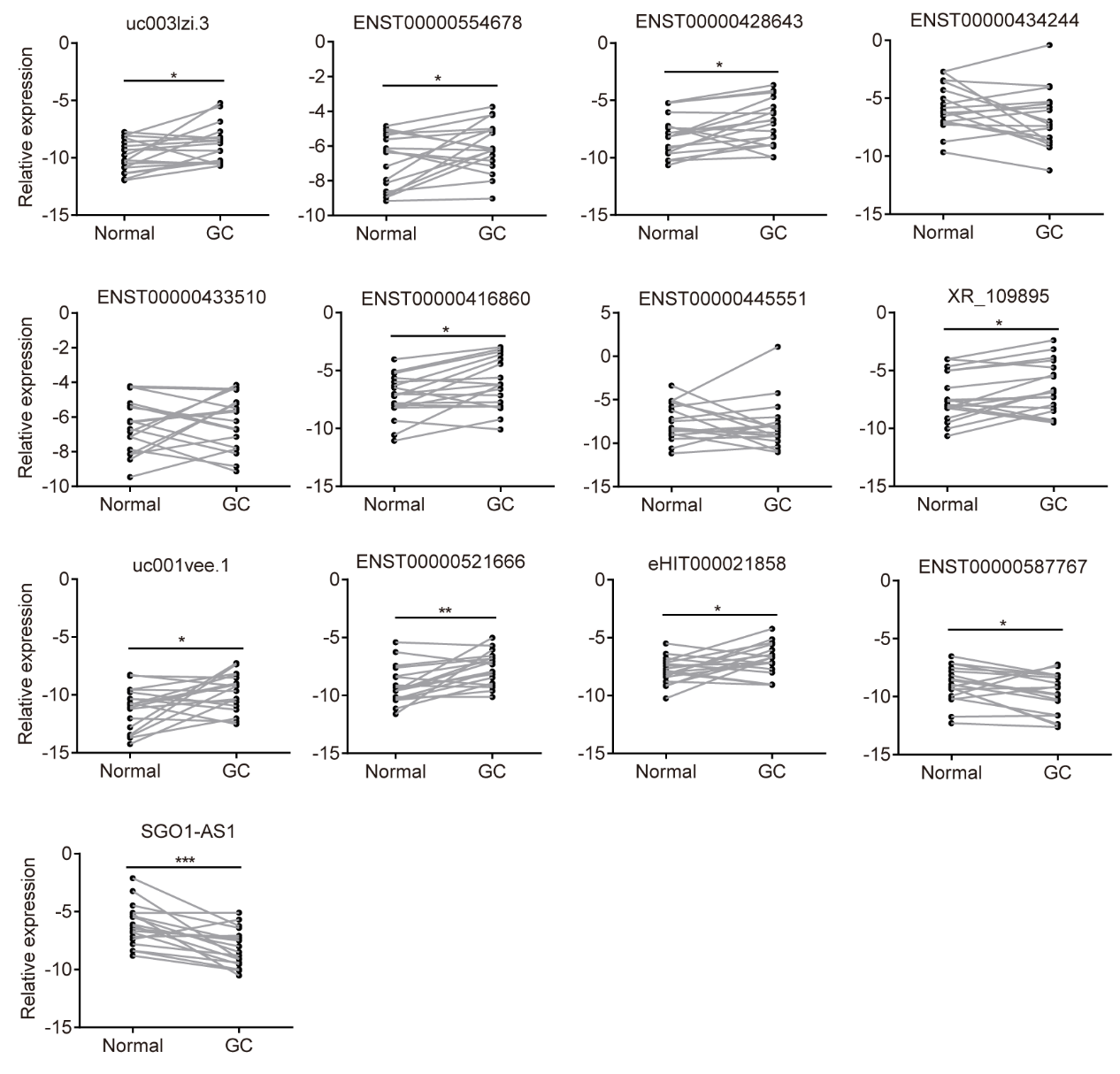


**Fig. S1** SGO1-AS1 was downregulated in GC tissues. Expression levels of SGO1-AS1 and 12 other lncRNAs validated by qRT-PCR in 18 paired gastric cancer tissues and adjacent normal tissues (Cohort 1). The results are expressed as -∆Ct. ***P* < 0.05, ***P* < 0.01, ****P* < 0.001.


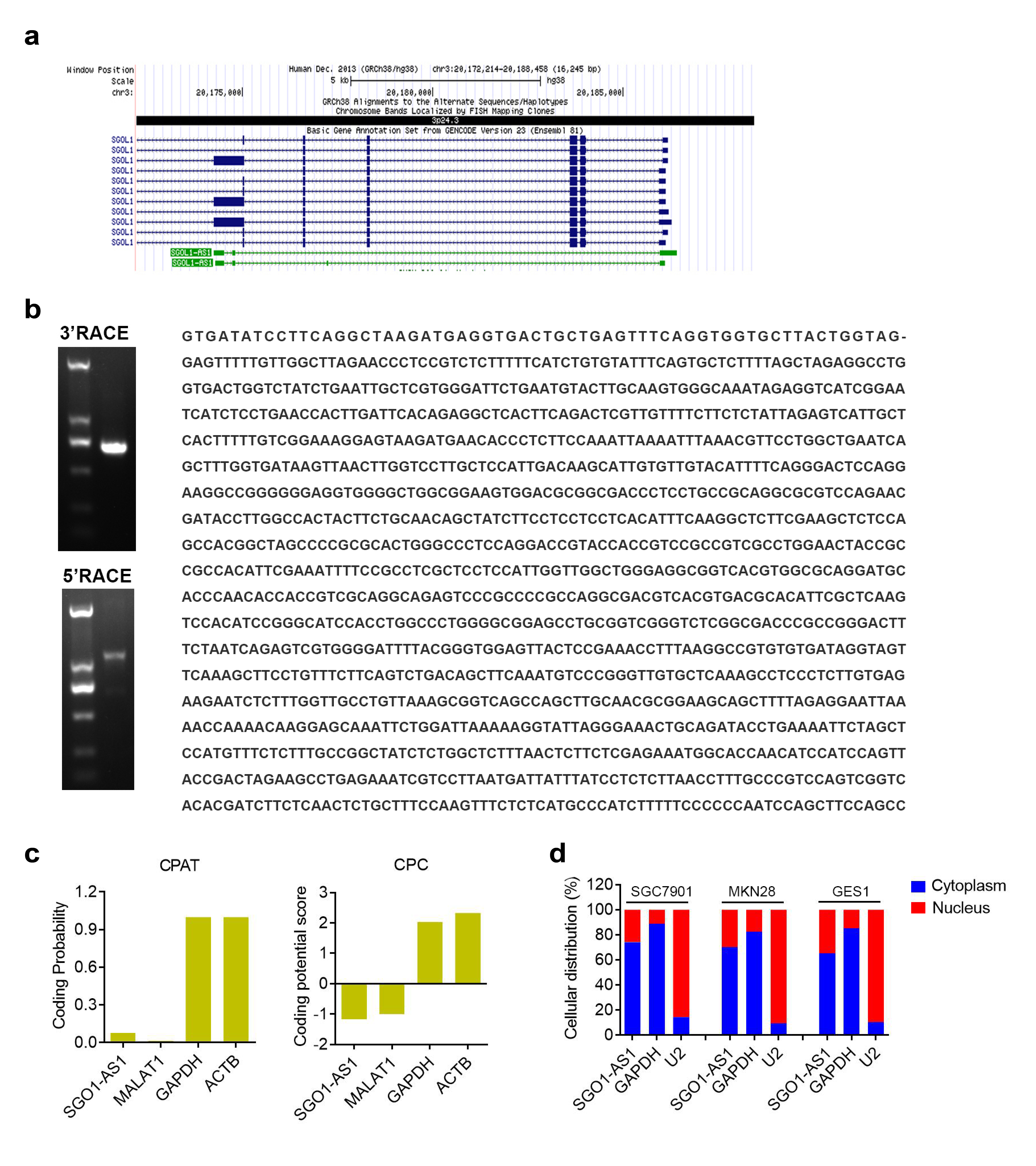


**Fig. S2** Characterization of human SGO1-AS1 as a long noncoding RNA. **a.** Schematic diagram of the genomic locus and isoforms of SGO1-AS1 in the UCSC Genome Browser (http://genome.ucsc.edu/). **b.** Identification of full-length SGO1-AS1 by 5’ and 3’ RACE. Left: Representative images of the PCR products from 5’ RACE and 3’ RACE. Right: Nucleotide sequence of full-length human SGO1-AS1. **c.** Coding potential of SGO1-AS1 as predicted by the CPAT and CPC tools. The lncRNA MALAT1 and the protein-coding genes GAPDH and ACTB are also shown. **d.** Relative distribution of SGO1-AS1 in gastric cell lines as determined by RT-PCR. SGO1-AS1 was mainly expressed in the cytoplasm of epithelial cells.


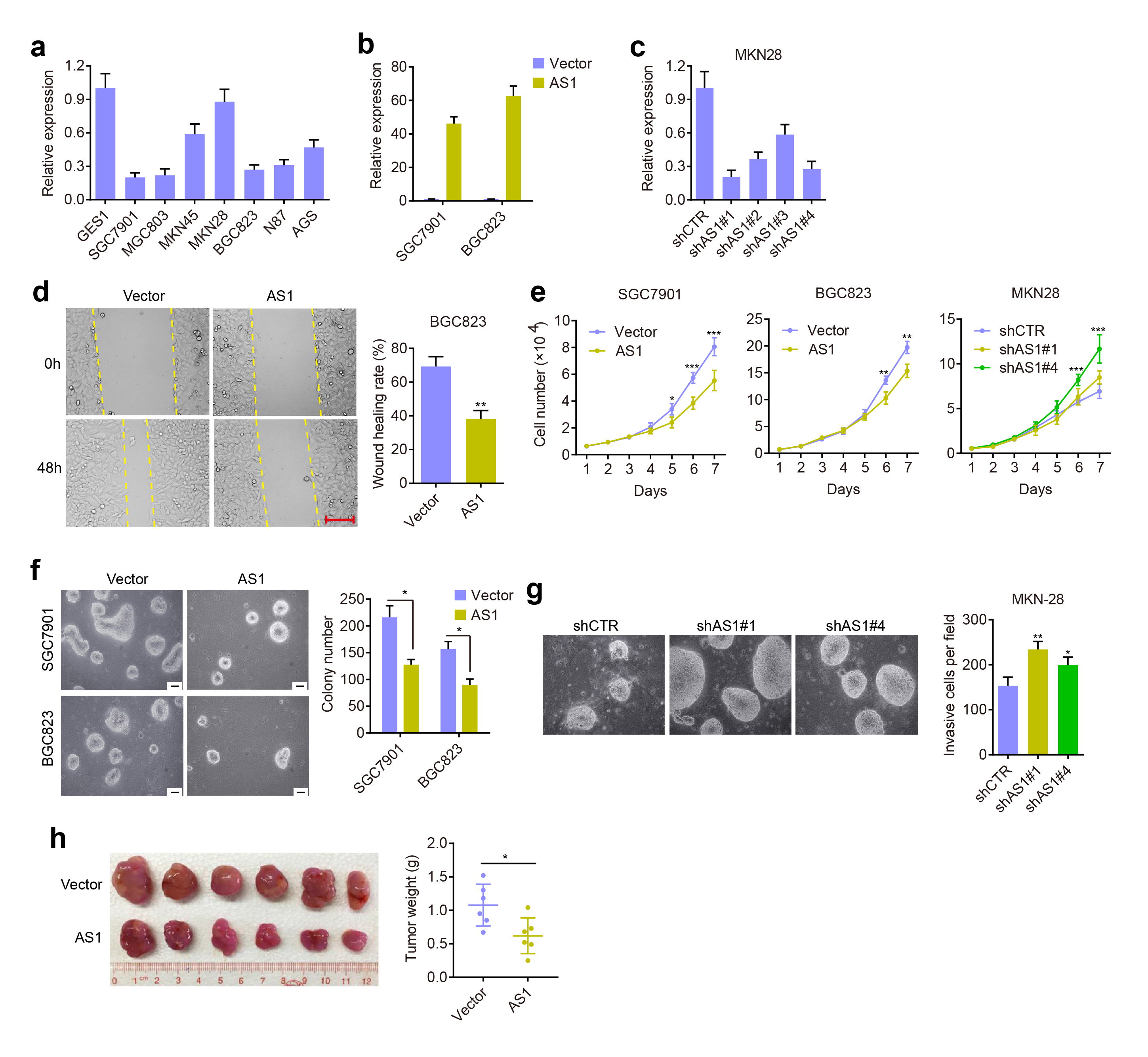


**Fig. S3** SGO1-AS1 inhibits GC cell migration and growth. **a.** qRT-PCR showed the relative levels of SGO1-AS1 expression in human gastric cancer cell lines compared to those in the immortalized human gastric epithelial cell line GES1. **b.** qRT-PCR analysis was used to confirm the level of SGO1-AS1 in cells with stable overexpression of SGO1-AS1. **c.** qRT-PCR analysis was performed to assess the inhibition efficiency in MKN28 cells infected with SGO1-AS1 shRNA lentiviruses targeting different regions of SGO1-AS1. **d.** Cell migration in BGC823 cells stably expressing SGO1-AS1 and control cells was examined by a wound healing assay. Scale bar, 150 μm. **e.** Cell proliferation assay in SGC7901 and BGC823 cells stably expressing SGO1-AS1, MKN28 cells stably silencing SGO1-AS1 and the respective control cells. **f-g.** A soft agar colony formation assay was carried out in the indicated cells. Scale bars, 100 μm. **h.** Overexpression of SGO1-AS1 inhibits GC tumor growth in a nude mouse model. SGC-7901 cells with stable overexpression of SGO1-AS1 or the control were inoculated subcutaneously into nude mice (n = 6 mice/group). The mice were sacrificed at 28 days postinoculation, and the tumors were weighed. Error bars indicate SD. **P* < 0.05, ***P* < 0.01, ****P* < 0.001.


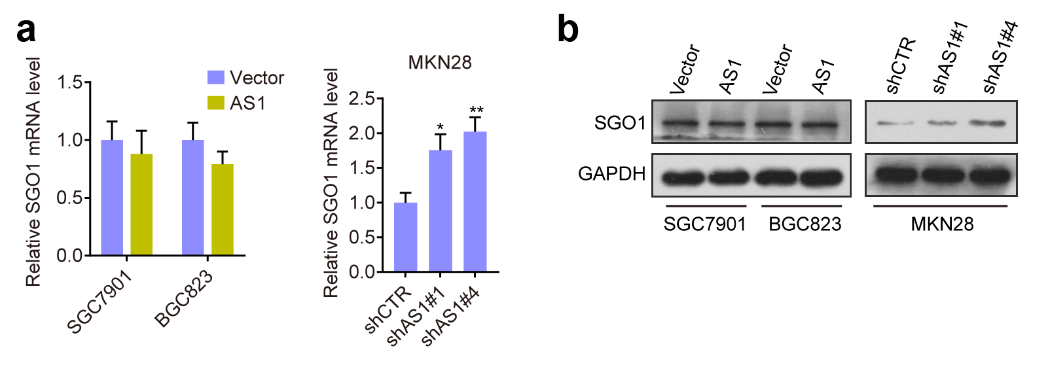


**Fig. S4** Effects of SGO1-AS1 on the expression of the sense gene SGO1. **a-b.** qRT-PCR and Western blotting analyses of the SGO1 levels in SGC7901 and BGC823 cells stably expressing SGO1-AS1, MKN-28 cells stably silencing SGO1-AS1 and the related control cells. Error bars indicate SDs from three independent experiments. **P* < 0.05, ***P* < 0.01.


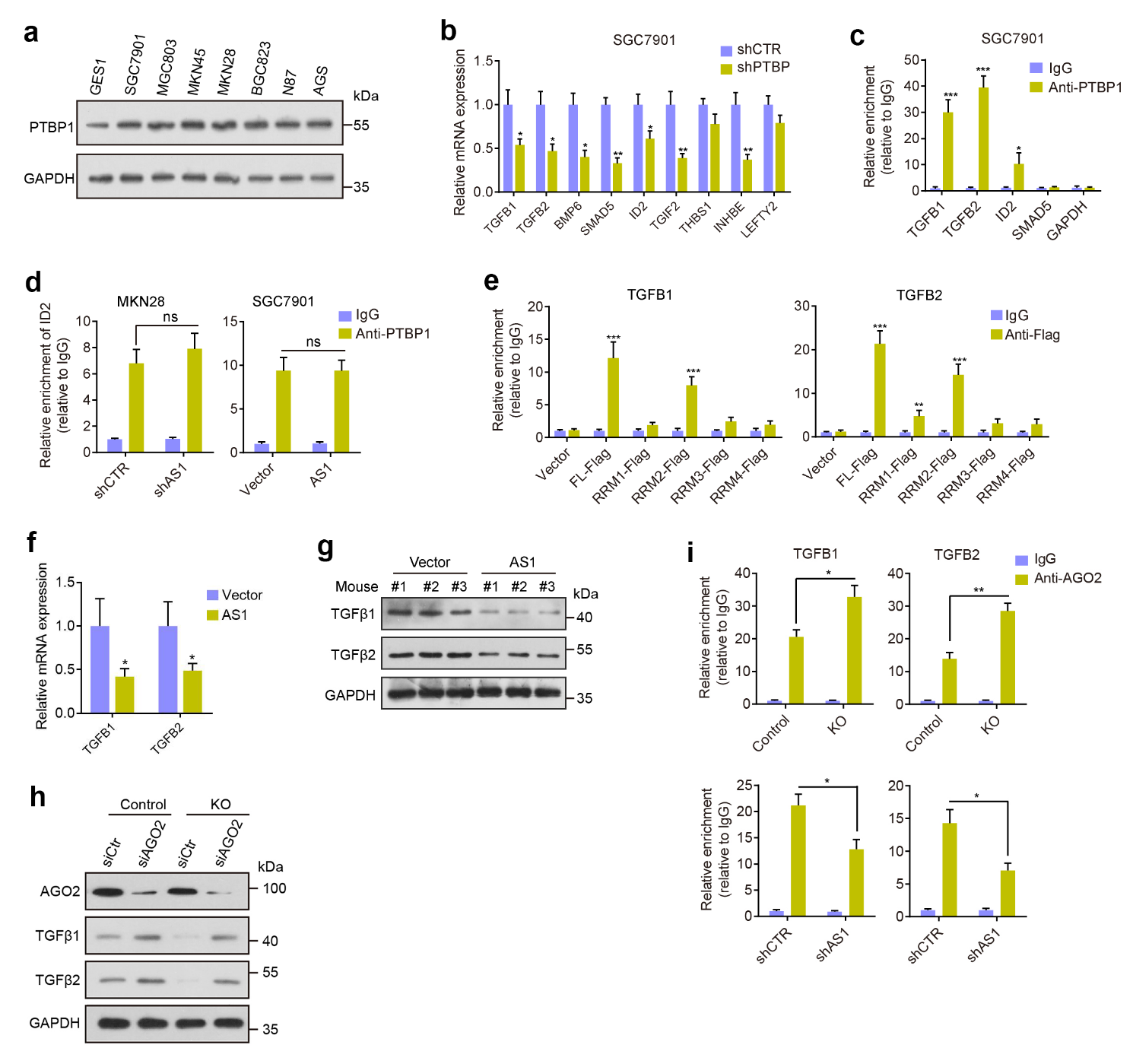


**Fig. S5** SGO1-AS1 reduces TGFB1/2 mRNA stability. **a.** Western blot analysis was used to evaluate PTBP1 expression in human gastric cancer cell lines and normalgastric epithelial cell line. **b.** qRT-PCR analysis was performed to examine the expression levels of genes related to the TGFβ pathway in SGC7901 cells transfected with PTBP1 shRNA or the control. **c.** PTBP1 RIP assay was performed to analyze the interactions between PTBP1 and TGFB1/2 or ID3 in SGC7901 cells. The relative fold enrichment of these mRNAs compared to IgG was determined by qRT-PCR. SMAD5 and GAPDH were used as negative controls. **d.** RIP-qPCR assay was used to evaluate the interaction between PTBP1 and ID2 mRNA in MKN28 cells with SGO1-AS1 knockdown or SGC7901 cells with SGO1-AS1 overexpression. **e.** Deletion mapping was performed to determine the binding domain of PTBP1 to TGFB1/2 mRNA using full-length or truncated PTBP1. **f-g.** mRNA **(f)** and protein **(g)** expression levels of TGFβ1/2 in xenograft tumor tissues recovered from nude mice. #1-3 denote individual tumors grown in different mice. **h.** Western blot analysis of AGO2, TGFβ1 and TGFβ2 in control and PTBP1-KO MKN28 cells transfected with siAGO2 or control siRNA. **i.** RIP-qPCR assay of the interaction between the AGO2 protein and TGFB1/2 mRNA in MKN28 cells with PTBP1 knockout or SGO1-AS1 knockdown. Error bars are SDs from three independent experiments. **P* < 0.05, ***P* < 0.01, ****P* < 0.001 and ns, not significant.


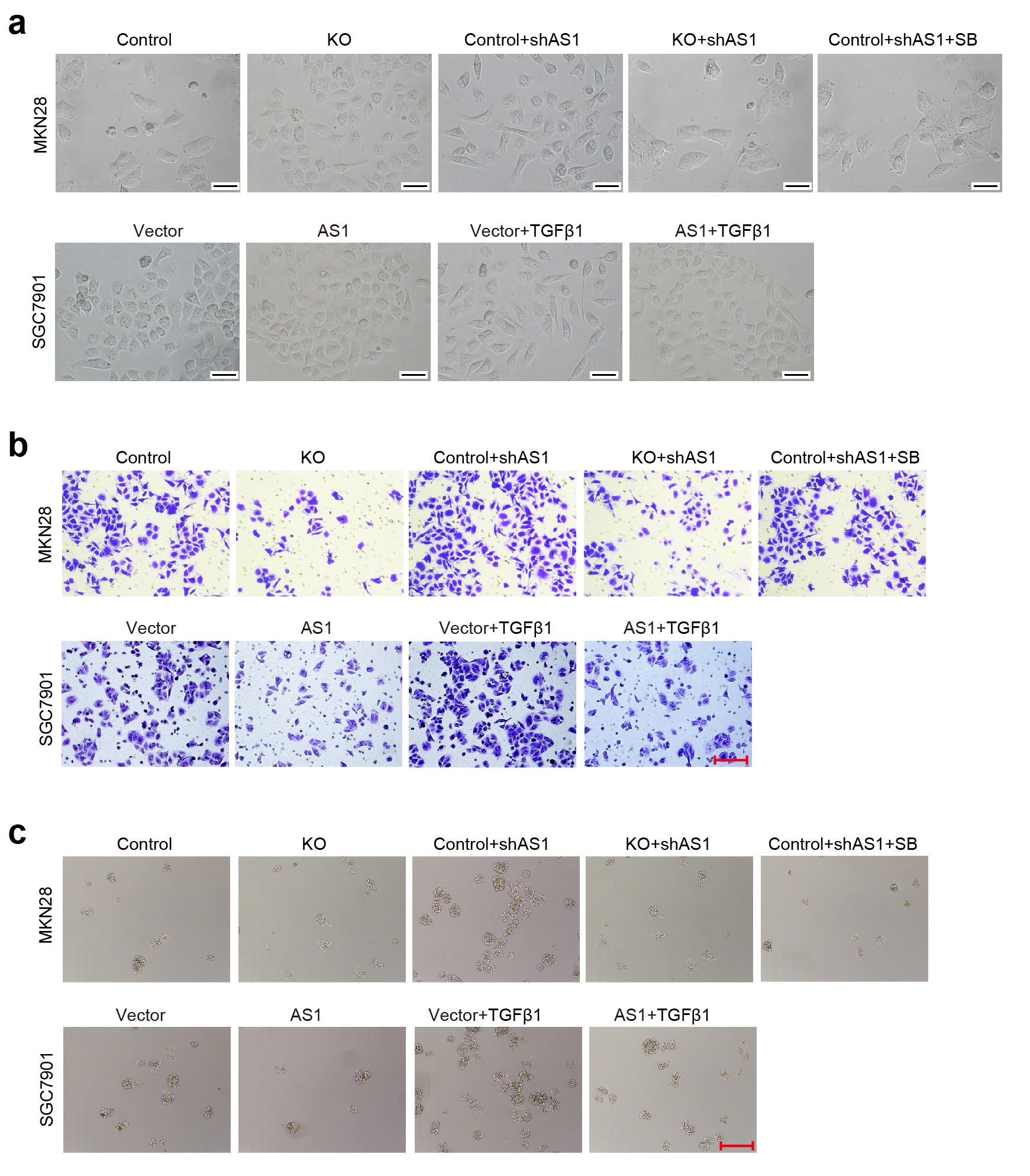


**Fig. S6** SGO1-AS1 changes cell morphology and suppresses cell invasion and stemness. **a-c.** Representative phase-contract **(a)**, cell invasion **(b)** and tumor spheroid images **(c)** of the indicated cells are shown. **a**, Scale bars,100μm; **b-c**, Scale bars, 150 μm.


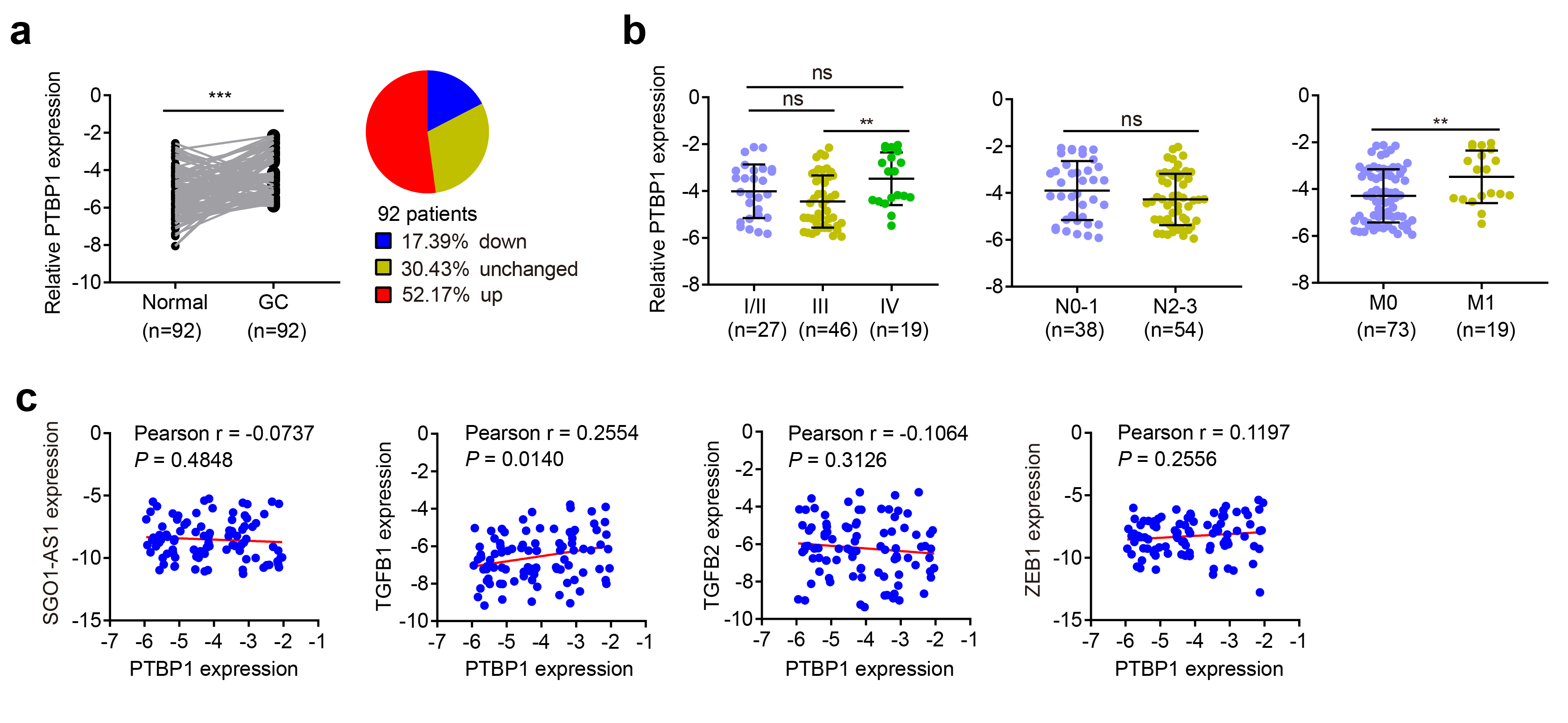


**Fig. S7** Expression level of PTBP1 and its correlation with SGO1-AS1, TGFB1/2 and ZEB1 expression in GC tissues. **a.** Relative expression levels of PTBP1 in 92 paired GC and normal tissues from Cohort 2 were quantified by qRT-PCR. The pie chart shows the proportions of samples in the downregulation (blue), upregulation (red) and no change (yellow) categories. **b.** The expression level of PTBP1 in GCs according to their clinical stage and status of lymph node or distant metastasis. **c.** Correlation between PTBP1 expression and SGO-AS1, TGFB1, TGFB2 and ZEB1 expression in GC specimens. a-c, Error bars indicate SDs. **P* < 0.05, ***P* < 0.01, ****P* < 0.001 and ns, not significant.

**Table S1.** Clinicopathological characteristics of Cohort 2 samples.

| **Characteristics** | **Cases** |
| --- | --- |
| Median age (range) | 59 years (35-82) |
| Gender  Male  Female | 62 (67.4%)  30 (32.6%) |
| Invasion depth  T1  T2  T3  T4 | 4 (4.3%)  9 (9.8%)  14 (15.2%)  65 (70.7%) |
| TNM stage  I  II  III  IV | 5 (5.4%)  22 (23.9%)  46 (50.0%)  19 (20.7%) |
| Lymph node metastasis  N0  N1  N2  N3 | 26 (28.3%)  12 (13.0%)  24 (26.1%)  30 (32.6%) |
| Distant metastasis  M0  M1 | 73 (79.3%)  19 (20.7%) |

**Table S2.** Clinicopathological characteristics of Cohort 3 samples.

| **Characteristics** | **Cases** |
| --- | --- |
| Median age (range) | 66 years (32-81) |
| Gender  Male  Female | 59 (62.1%)  36 (37.9%) |
| Tumor size  <5cm  ≥5cm | 42 (44.2%)  53 (55.8%) |
| Invasion depth  T1  T2  T3  T4 | 8 (8.4%)  11 (11.6%)  57 (60.0%)  19 (20.0%) |
| TNM stage  I  II  III  IV | 10 (20.0%)  30 (31.6%)  48 (50.5%)  7 (7.4%) |
| Lymph node metastasis  N0  N1  N2  N3 | 25 (26.3%)  15 (15.8%)  26 (27.4%)  29 (30.5%) |
| Distant metastasis  M0  M1 | 73 (79.3%)  19 (20.7%) |

**Table S3.** Primers used for quantitative PCR

| **Gene** | **Forward primer sequence (5′→3′)** | **Reverse primer sequence (5′→3′)** |
| --- | --- | --- |
| SGO1-AS1 | CCGTTTTTCATCTGTGTATT | CCGGAAGAGCCTTGAAATGT |
| GAPDH | AATGGGCAGCCGTTAGGAAA | GCCCAATACGACCAAATCAGAG |
| ACTB | AGCACAGAGCCTCGCCTTT | ATCATCATCCATGGTGAGCTGG |
| TGFB1 | AACCCACAACGAAATCTATGAC | GCTGAGGTATCGCCAGGAAT |
| TGFB2 | CTACTTAATAGCCACTCGTC | CTAGTCAATGCCCAACAG |
| BMP6 | TCATTCCCAGAAGTCCACA | ACTCCAGCACGAACATACAA |
| SMAD5 | ACTAGGCGACATATTGGA | CTTACAGACAGTGGTGGG |
| ID2 | CCCCAGAACAAGAAGGTG | ATCCGTGTTGAGGGTGGT |
| TGIF2 | CACCGCTACAACGCCTAC | GGTCTTTGCCATCCTTCC |
| THBS1 | TCCTGCGATAGCCTCAAC | CTCCAGCCACCATCCTGT |
| INHBE | ATTGCTGCCTCTTTCCATT | CATCCGTCTTGACCACATT |
| LEFTY2 | CTGTGACCCTGAAGCACCAAT | GGCAGTCTCCGAGGCGATA |
| ZEB1 | AATCATCGCTACTCCTACT | TCTTCCCTTGTCAAACTC |
| ZEB2 | ACCCCCAATAAATATAGGACTGGA | GAGAAGGCAACTGGACCGAA |
| TWIST1 | TCGGTCTGGAGGATGGAGGG | GGGAACCTGTAACTGTGCGTGA |
| SNAI | TTACCTTCCAGCAGCCCTAC | AGCCTTTCCCACTGTCCTC |
| SLUG | CAGCTCAGGAGCATACAG | GAGGAGGTGTCAGATGGA |
| SGO1 | TCCAGGACAAGGAGAATC | TCAGGCTAAGATGAGGTG |
| PTBP1 | TTTTCCAAGCTCACCAGCCT | TATACCAGGTGCACCGAAGG |
| PTBP2 | GCTTCCTGTTGCAGCTGTTC | AGGACTGTATTGCCACCAGC |
| PTBP3 | CCTTGAACCCCCTATGGCTG | AGGAAATCCAATGGCTGGGG |
| G3BP2 | CCGGCAGAACCTGTTTCTCT | ACACGAGGTGGCTGAGATTG |
| FUBP3 | TCCGGCAGATTGCTGCTAAA | AGGCCCCTAACTGGTTACCT |
| HNRNPK | TGCGAGTTGAGGCTGTTGAT | GCACGTCCTTTGATGGGAGA |
| HNRNPM | TGGACGCTGAAGGAAAGTCA | AATCATTCCTGGGCCACCTG |
| SGO1-AS1 promoter Primer 1 | CAAAAAAGTCCCTCTGTCC | TACTTACCATCTGTTTCTCTG |
| SGO1-AS1 promoter Primer 2 | TCACCATGTTGGCCAGGCTG | ACAATTCTGTGAAGTATAGG |

**Table S4.** Primers for rapid amplification of cDNA ends analysis

| **Primer Name** | [**Sequence**](D:/program%20files/Youdao/Dict/8.5.1.0/resultui/html/index.html#/javascript:;)**(5′→3′)** |
| --- | --- |
| SGO1-AS1-5’-outer- reverse | ATCGTGTGACCGACTGGACG |
| SGO1-AS1-5’-inner- reverse | TGGTGCCATTTCTCGAGAAGA |
| SGO1-AS1-3’-outer- forward | CCGTACCACCGTCCGCCGTCG |
| SGO1-AS1-3’-inner- forward | TTGGTTGGCTGGGAGGCGGTCA |

**Table S5.** Correlation between clinicopathological parameters and SGO1-AS1 levels in 95 cases of GC tissues (Cohort 3).

| **Viable** | **All cases** |  | **SGO1-AS1** | | |
| --- | --- | --- | --- | --- | --- |
|  |  |  | **Low** | **High** | ***P* value** |
| Age (years)  ≤65  >65 | 47  48 |  | 27 (57.4%)  27 (56.3%) | 20 (42.6%)  21 (43.8%) | 0.906 |
| Gender  Male  Female | 59  36 |  | 33 (55.9%)  21(58.3%) | 26 (44.1%)  15 (41.7%) | 0.819 |
| Tumor size  <5cm  ≥5cm | 42  53 |  | 18 (42.9%)  36 (67.9%) | 24 (57.1%)  17 (32.1%) | **0.014** |
| Invasion depth  T1/2  T3/4 | 19  76 |  | 6 (31.6%)  48 (63.2%) | 13 (68.4%)  28 (36.8%) | **0.013** |
| TNM stage  I/II  III/IV | 40  55 |  | 14 (35.0%)  40 (72.7%) | 26 (65.0%)  15 (27.3%) | **<0.001** |
| Lymph node metastasis  Absent  Present | 25  70 |  | 10 (40.0%)  44 (62.9%) | 15 (60.0%)  26 (37.1%) | **0.048** |

**Table S6.** Univariate and multivariate analyses of factors associated with overall survival.

| **Parameters** | **Univariate analysis** | |  | **Multivariate analysis** | |
| --- | --- | --- | --- | --- | --- |
|  | **HR** | ***P* value** |  | **HR** | ***P* value** |
| Age (>65 vs.≤65) | 1.922 (1.163-3.178) | 0.011 |  | 2.014 (1.225-3.310) | 0.006 |
| Gender (female vs. male) | 1.095 (0.666-1.799) | 0.721 |  | / | / |
| Tumor size (≥5cm vs.<5cm) | 1.591 (0.933-3.883) | 0.088 |  | 1.603 (0.944-2.273) | 0.080 |
| Invasion depth (T3/4 vs. T1/2) | 1.494 (0.681-3.276) | 0.371 |  | / | / |
| TNM stage (III/IV vs. I/II) | 1.633 (0.687-3.883) | 0.267 |  | 1.819 (1.041-3.180) | 0.036 |
| Lymph node metastasis  (present vs. absent) | 1.007 (0.417-2.436) | 0.987 |  | / | / |
| SGO1-AS1 expression  (high vs. low) | 0.522 (0.305-0.894) | 0.018 |  | 0.510 (0.300-0.867) | 0.013 |

**Table S7.** Mass spectrometry protein identification results for biotinylated

SGO1-AS1 RNA pull down

| **Protein** | **prot_score** | **prot_sequences** | **prot_sequences_sig** |
| --- | --- | --- | --- |
| PTPB1 | 4304 | 23 | 21 |
| PTBP3 | 1488 | 12 | 10 |
| PTBP2 | 1412 | 18 | 15 |
| HNRNPK | 376 | 14 | 11 |
| NCL | 352 | 17 | 8 |
| HNRNPM | 298 | 16 | 11 |
| FUBP3 | 249 | 16 | 8 |
| SYNCRIP | 232 | 10 | 5 |
| CPSF7 | 200 | 12 | 5 |
| G3BP2 | 156 | 11 | 7 |
| ALB | 149 | 15 | 5 |
| PRPF4 | 132 | 8 | 6 |
